# In-depth insights into Alzheimer’s disease by using explainable machine learning approach

**DOI:** 10.1038/s41598-022-10202-2

**Published:** 2022-04-20

**Authors:** Bojan Bogdanovic, Tome Eftimov, Monika Simjanoska

**Affiliations:** 1 Faculty of Computer Science and Engineering, Ss. Cyril and Methodius University, Skopje, 1000 North Macedonia; 2 Computer Systems Department, Jozef Stefan Institute, Ljubljana, 1000 Slovenia; 3 iReason, LLC, Skopje, 1000 North Macedonia

**Keywords:** Data processing, Databases, Machine learning, Predictive medicine, Statistical methods, Alzheimer's disease

## Abstract

Alzheimer’s disease is still a field of research with lots of open questions. The complexity of the disease prevents the early diagnosis before visible symptoms regarding the individual’s cognitive capabilities occur. This research presents an in-depth analysis of a huge data set encompassing medical, cognitive and lifestyle’s measurements from more than 12,000 individuals. Several hypothesis were established whose validity has been questioned considering the obtained results. The importance of appropriate experimental design is highly stressed in the research. Thus, a sequence of methods for handling missing data, redundancy, data imbalance, and correlation analysis have been applied for appropriate preprocessing of the data set, and consequently XGBoost model has been trained and evaluated with special attention to the hyperparameters tuning. The model was explained by using the Shapley values produced by the SHAP method. XGBoost produced a f1-score of 0.84 and as such is considered to be highly competitive among those published in the literature. This achievement, however, was not the main contribution of this paper. This research’s goal was to perform global and local interpretability of the intelligent model and derive valuable conclusions over the established hypothesis. Those methods led to a single scheme which presents either positive, or, negative influence of the values of each of the features whose importance has been confirmed by means of Shapley values. This scheme might be considered as additional source of knowledge for the physicians and other experts whose concern is the exact diagnosis of early stage of Alzheimer’s disease. The conclusions derived from the intelligent model’s data-driven interpretability confronted all the established hypotheses. This research clearly showed the importance of explainable Machine learning approach that opens the black box and clearly unveils the relationships among the features and the diagnoses.

## Introduction

Alzheimer’s disease (AD) is considered to be common cause of dementia worldwide^1^. Over time, people with Alzheimer’s disease suffer memory loss as well as the ability to concentrate. In advanced stages of the disease, there are severe complications such as dehydration, malnutrition or infection, which eventually result in death^2^. Since its first description in the early twentieth century, there is still no treatment that cures Alzheimer’s disease or alters the disease process in the brain. However, the course of Alzheimer’s can be positively influenced by a number of different medications and non-drug treatments, making it crucial for people with Alzheimer’s to receive early good care and support.

Even associated with forgetfulness, AD affects different aspects of individual’s personality, life experiences, current circumstances and wrong responses to the situations they suddenly find themselves in^3^, and relationships with other people as is the verbal communication. The gradual nature of affecting the short-term memory at first and the long-term memory at later stages makes the maintenance of orientation in time and space inevitably difficult. This is also visible through the work of an artist that presents a time-series of self-portraits of his Alzheimer’s disease progresses in time^4^ and the original work clearly shows the cognitive decline and spatial disorientation, however, the emotion still highly remains in each of the original works.

It is not yet possible to diagnose Alzheimer’s with complete certainty using the currently available tests while the person is still alive. The disease is diagnosed if someone has the typical symptoms eliminating all the other possible causes. Since symptoms like forgetfulness, changes in behavior and problems with orientation might have many different causes, it is important not to rush to a diagnosis of Alzheimer’s. The symptoms might also be caused by depression or other physical conditions like meningitis, a stroke or bleeding in the brain^5^. Conducting an effective clinical trial is crucial to accurately predict the change in AD’s indicators so that the effect of the treatment can be assessed. The most common approaches are:

- Manual prediction by a clinical expert by using the clinical history of patients with similar conditions and visual analysis of various brain scans.
- Regression analysis to predict the future indicator changes in patient status, based on data from MRI^6^, cognitive test scores^7^, rate of cognitive decline^8^, and also retrospectively staging subjects by time to conversion between diagnoses^9^.
- Supervised Machine Learning (ML) has already shown to be effective in discrimination between AD patients from cognitively normal subjects by using MR images^10^, variety of biomarkers^11^, etc.
- Data-driven disease progression models are most recently used to predict AD in unsupervised manner. Examples include models built on a set of biomarkers to produce discrete^12,13^ or continuous^14,15^ pictures of disease progression. Also there are less comprehensive models that leverage structure in data such as MR images^16,17^.

Even though it is still unclear what is the main cause of the disease, it has been shown that people with Alzheimer’s do not have enough of an important chemical messenger called **acetylcholine** in their brain^18^. And it has also been shown that small protein particles (*for example plaques*) build up in their brain. These might cause the nerve cells to die^19^.

In^20^ several factors have been hypothesized to play a role in the Alzheimer’s disease occurrence, and those are:

- *Age*–starting at about age of 65, the probability of getting AD doubles every 5 years^21,22^.
- *apolipoprotein E4 (APOE E4)*–10 to 30 times higher of developing AD compared to non-carriers, i.e., subjects without the gene. However the exact mechanism through which the presence of APOE E4 leads to AD is not known^23^.
- *Gender*–women seem more likely to develop AD than men. The reasons for this are still unclear^24^.
- *Medical conditions*–type 2 diabetes, high blood pressure, high cholesterol, obesity^25^, or depression^26^ are known to increase the risk of developing dementia.
- *Lifestyle factors*–physical inactivity^27^, smoking^28^, unhealthy diet^29^, excessive alcohol^30^, or head injuries^31^.

Newest clinical researches provide contemporary view about differentiating clinically diagnosed AD dementia from other neurodegenerative disorders using plasma P-tau217^32^, improvements in neuropathological diagnosing the disease^33^ and possible approach for a drug development against its progression^34^.

Considering the complexity of Alzheimer’s disease and the fact that multiple factors under various circumstances affect the onset of it, it is not sufficient to do simple machine learning experiments and aim for the best metrics. Using the interpretability of the ML model, provided by various explainable machine learning techniques, can significantly help obtaining a bigger picture about risk factors influences on the particular diagnosis. The interpretability shows interesting yet still not proven trends that are present in the used dataset.

Multiple recent papers using interpretability techniques have provided compelling results and guidelines^35^ for further medical expertise, including regular^36^ and multi layer multi modal^37^ interpretability of the Alzheimer’s disease, interpretability of ensemble learning algorithms for predicting dementia^38^ and extracting explainable assessments from MRI imaging scans^39^.

### Hypothesis

This research is focused on deep investigation of some of the factors that are claimed to play an important role for the occurrence and further development of Alzheimer’s disease by following explainable Machine Learning (ML) approach. The research puts the following hypothesis at test:

- **Hypothesis 1**: There is gender predisposition for obtaining Alzheimer’s disease.
- **Hypothesis 2**: APOE4 gene is crucial decisive factor for Alzheimer’s disease diagnosis.
- **Hypothesis 3**: Older people are more prone to Alzheimer’s disease.
- **Hypothesis 4**: Cognitive tests distinguish between all stages leading to Alzheimer’s disease.

To test the established hypothesis, a data set encompassing 12,741 subjects medically confirmed to belong into five categories - cognitively normal (CN), Early Mild Cognitive Impairment (EMCI), Late Mild Cognitive Impairment (LMCI), Significant Memory Concern (SMC) or Alzheimer’s disease (AD), will be used to develop intelligent model able to classify the patients with high precision and recall with the aim to further interpret the model by the explainable ML methods.

The paper is organized as follows. The data set is fully described in “Materials and methods” section. In the same “Materials and methods” section there is a comprehensive description of the data preprocessing as well as the ML approach applied to the data set. The intelligent model’s results are provided in “Results” section. The model is interpreted by means of explainable ML in “Explainable machine learning” section upon which a discussion showing the importance of the research is provided in “Discussion” section. “Conclusion” section presents the final conclusion over the established hypotheses.

## Materials and methods

**Figure 1:**
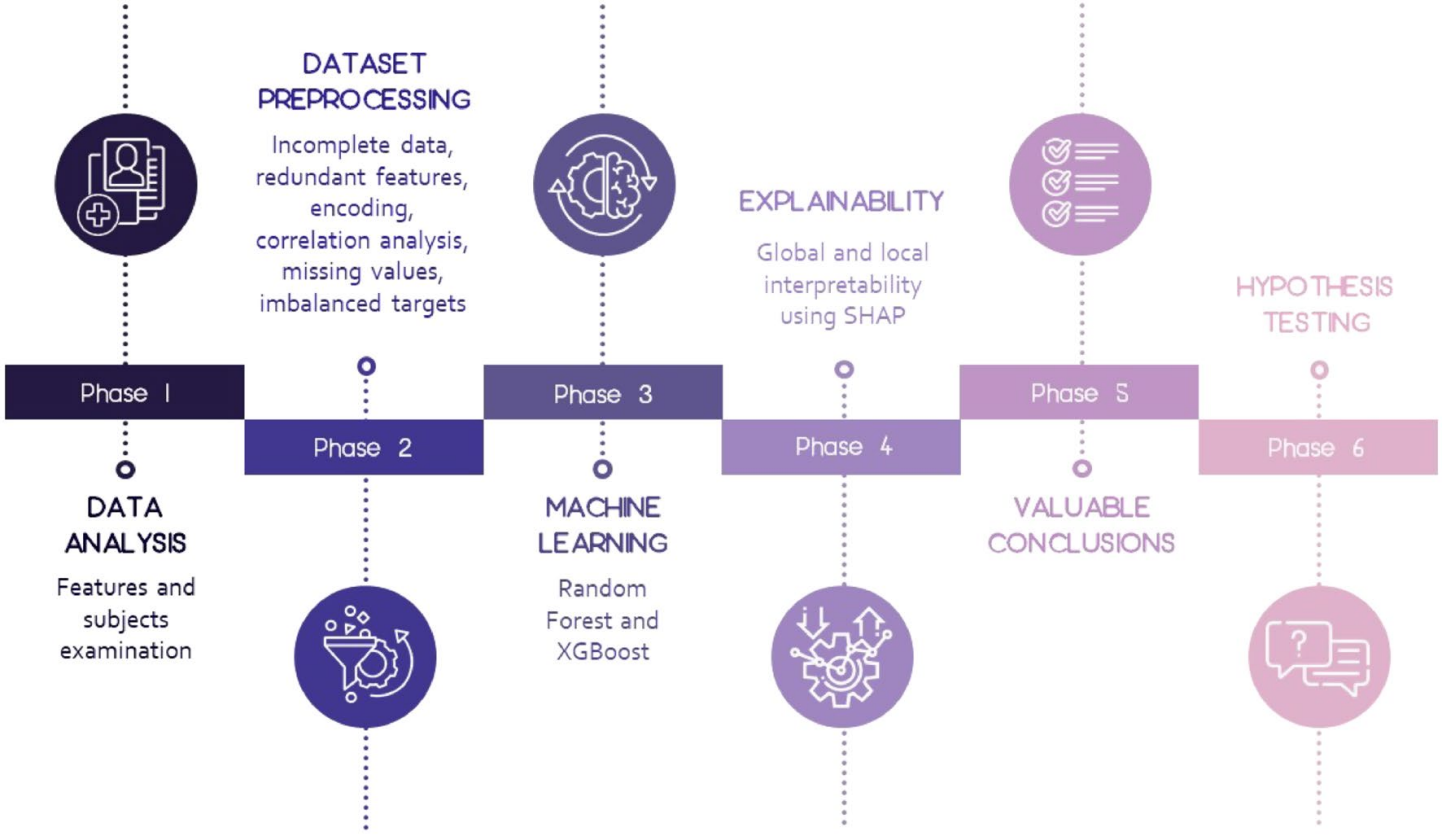
Methodology workflow.

Figure 1 presents the complete methodology as a roadmap followed to analyse and derive valuable conclusions from the data. Six different phases can be distinguished encompassing methods as follows:

- Phase 1: Data set analysis. In this step the subjects and all the features available in the database are analysed in terms of their importance for the problem at hand.
- Phase 2: Data set preprocessing. This phase is focused on methods for incomplete data, redundant features, encoding, correlation analysis, missing values, and imbalanced targets handling, from which as a result a data set prepared for ML will be obtained.
- Phase 3: Training ML model. At the ML phase, XGBoost model has been trained, and also hyperparameters tuning has been performed.
- Phase 4: Interpretability. This is the most important phase considering the contribution of the research, since it allows the global and local interpretability of the trained model by using Shapley values and thus, provides deep insight into the features influence on the prediction of each of the classes.
- Phase 5: Valuable conclusions. Based on the previous phase, valuable conclusions are derived at this phase followed by comprehensive discussion.
- Phase 6: Hypothesis testing. At the last phase, the research is concluded by reconsidering the established hypothesis based on the results from Phase 5.

### Data set analysis

The data set used in this research is used in the TADPOLE Challenge (The TADPOLE Challenge compares the performance of algorithms at predicting the future evolution of individuals at risk of Alzheimer’s disease.) and retrieved from **ADNI** (*Alzheimer’s Disease Neuroimaging Initiative*) available at their official website (*http://adni.loni.usc.edu/*). In order to obtain permission for data usage, a request has been sent together with an explanation for the purpose of the research.

The original data set contains data from 12,741 subjects and 1,907 attributes. Mainly the attributes arise from two categories: quantitative parameters and personal information. As provided in the data set description, the data is labeled by medical experts.

Considering the sparse data set problem and according to the suggestions provided in the data set source^40^, 17 features have been chosen to be informative for most of the patients covering personal information, gene expression analysis, medical information and cognitive tests information. Those feature are considered to carry information needed to test the hypothesis established in “Hypothesis” section.

Each row represents data for one subject, and each column represents a feature or measurement related to the subject. The features can be divided into six categories, encompassing:

- *Personal information*:
  - *PTID*: Participant ID
  - *AGE*: Age at baseline
  - *PTGENDER*: Sex
  - *PTEDUCAT*: Years of education
  - *PTRACCAT*: Race
- *Gene expression*:
  - *APOE4*: Expression of the ApoE4 gene
- *PET measures*:
  - *FDG*: measure cell metabolism, where cells affected by AD show reduced metabolism.
  - *AV45*: measures amyloid-beta load in the brain, where amyloid-beta is a protein that mis-folds (*i.e. its 3D structure is not properly constructed*), which then leads to AD.
- *MRI measures*:
  - *Hippocampus*: scan of a complex brain structure embedded deep into temporal lobe.
  - *WholeBrain*: scan of the subject’s whole brain.
  - *Entorhinal*: scan of an area of the brain that is located in the medial temporal lobe and functions as a hub in a widespread network for memory, navigation and the perception of time.
  - *MidTemp*: scan of the middle temporal artery.
- *Cognitive tests*:
  - *CDRSB*: Clinical Dementia Rating Scale - Sum of Boxes.
  - *ADAS11*: Alzheimer’s Disease Assessment Scale 11.
  - *MMSE*: Mini-Mental State Examination.
  - *RAVLT_immediate*: Rey Auditory Verbal Learning Test (sum of scores from 5 first trials).
- *Target*:
  - *DX_bl*: Subject’s diagnosis, i.e., the **target variable** of which we want to gain a deeper understanding. We built models whose goal is to predict the value of this variable based on the values of other features. The target variable can result in any of the following five values: **CN** (*Cognitive Normal*), **EMCI** (*Early Mild Cognitive Impairment*), **LMCI** (*Late Mild Cognitive Impairment*), **SMC** (*Significant Memory Concern*) and **AD** (*Alzheimer’s Disease*).

Those features are measured by techniques that are able to assess some indicators of whether the individual might be at risk of development, or, has already developed AD symptoms.

The cognitive tests allow the examiner to obtain an overall sense of whether a person is aware of the symptoms, the surrounding environment, whether he/she can remember a short list of words, follow some instructions and do simple calculations. Cognitive tests are able to measure cognitive decline in a direct and quantifiable manner. However, the cognitive decline is one of the latest to become abnormal. This is because the first abnormalities are first noticed at a microscopical scale through the misfolding of a protein called Amyloid beta. These are followed by changes at larger scales, such as loss of the neurons myelin sheath, neuron death, visible atrophy in MRI scans and finally cognitive decline.

Cognitive tests, however, have several limitations that affect their reliability and those are related to remembering them if taken several times, might have floor or ceiling effects, which means that many subjects might score the highest/lowest score possible, and can be biased, as they are undertaken by a human expert who might be influenced by prior knowledge of the subject’s cognitive abilities^41^.

Magnetic resonance imaging (MRI) is a technique used to quantify by measuring the volume of **gray matter - GM** (consisted of nerve cells) and **white matter - WM** (fibres connecting the nerve cells). **Atrophy** is indicated by the loss of volume between one scan and other follow-up scan. It is caused by the death of neurons in regions affected. Quantification of atrophy with MRI is a very important parameter as it is widely available and non-invasive good indicator of progression of MCI to dementia^42–44^.

The Positron Emission Tomography (PET) enables researchers to track the concentration of abnormal proteins (amyloid and tau) since the contrast agent (containing the tracer) spreads throughout the brain and binds to abnormal proteins. PET scans can be of several types, depending on the cellular and molecular processes that are being measured. Fluorodeoxyglucose (FDG) PET can be used to measure **cell metabolism**. Neurons that are about to die show reduced metabolism, so FDG PET is an indicator of neurodegeneration. AV45 PET is used to measure the levels of abnormal proteins such as **amyloid-beta**.

The errors in Amyloid-beta 3D structure (misfolding) is thought to be one of the causes of AD since its high levels lead to neurodegeneration and cognitive decline. The basic limitation of PET scans is that the patient is exposed to ionizing radiation, which limits the number of scans they can take in a specific time interval^42,45^.

The **APOE gene** provides instructions for producing a protein called apolipoprotein E, and that is why the gene expression measurement is an important technique by which the activity of APOE gene can be quantified. This protein aids the formation of lipoproteins by combining with fats (*lipids*) in the body. There are at least three slightly different versions (alleles) of the APOE gene. The major alleles are e2, e3, and e4. The **e4 version** of the APOE gene is believed to increase an individual’s risk for developing AD. People who inherit one copy of the APOE e4 allele have an increased chance of developing the disease, those with two copies of the allele are at even greater risk. In TADPOLE data set, an information about individual’s number of present e4 alleles (0, 1 or 2) is available. However, not necessarily the individuals with AD have the APOE e4 allele, and also not all individuals who have this allele will develop AD^46^.

### Data set preprocessing

#### Incomplete data

**Figure 2:**
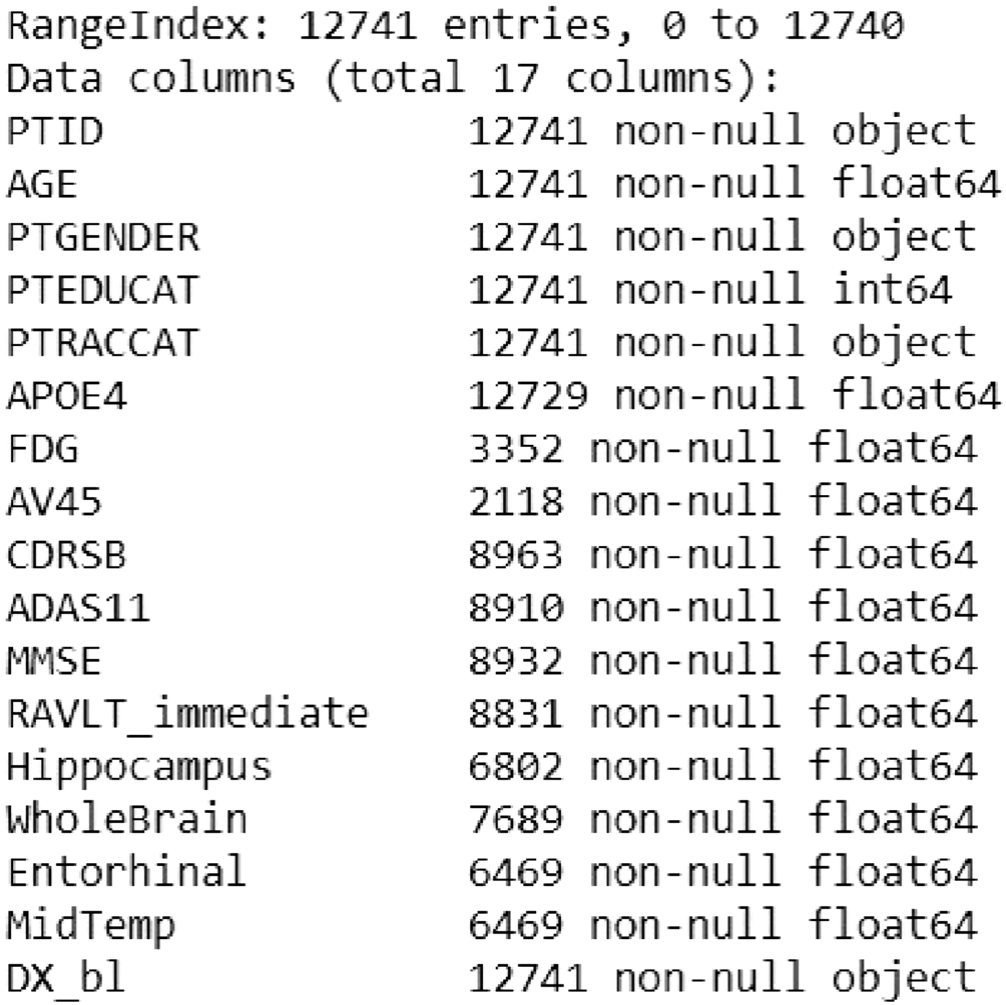
Data set summary.

Figure 2 shows that all subjects contain personal information and diagnosis, but not all of them have data for all the other parameters. All subjects with incomplete data have been detected since incomplete data can cause a lot of troubles in the process of data analysis and building the intelligent model later described in this section. Since the removal of all subjects where data for at least one attribute is missing causes significant information loss, an attempt is be made to use the leverage of some imputation techniques.

For example, only 2,118 subjects have data for the AV45 attribute. Removing all subjects (rows) that contain any missing data from our data set results into new data set with 1,121 entries.

Therefore, it is decided to remove all subjects that miss data for more than 6 features, and then perform data augmentation by using the imputation techniques later described in this section. The initial data removal resulted in 9,592 subjects left for further analysis.

#### Redundant features

The redundancy analysis of the features is crucial step for appropriate experimental setup. Taking a closer look at the PTRACCAT column in Table 1, it can be perceived that almost 93% entries have value ’white’. It means that this attribute does not provide enough information about possible racial predisposition for the disease. Most of the subjects belong to same racial group and only few belong to other groups, so this feature is be excluded from further analysis. Also PTID attribute is omitted because it is an identification number for each subject and has no meaning for the models we intend to build.

**Table 1:** Unique PTRACCAT values (only subjects with non missing data are considered).

Race	Number of subjects
White	1046
Black	36
More than one	16
Asian	15
Unknown	3
Hawaiian/Other PI	3
Am Indian/Alaskan	2

#### Categorical features encoding

The attributes **PTGENDER** (Male/Female) and **DX_bl** (CN, EMCI, LMCI, SMC, and AD) are of categorical data type. Simply encoding the attribute ’Male’ with the value 1 while ’Female’ with 0, would lead to increase the weight of Male compared to that of Female. This does not make sense since both variables need to be treated equally by the model to predict accurate results. To achieve equality one-hot encoding is used for encoding the PTGENDER attribute. This encoding is appropriate for categorical data where no relationship exists between categories. It involves representing each categorical variable with a binary vector that has one element for each unique label and marking the class label with a 1 and all other elements 0.

Considering the target attribute **DX_bl**, it can be seen that there is a certain order related to its values. The values can be ranked from CN to AD, based on the subject’s neuropsychological disorder. Therefore, Label encoding has been used to simply convert labels to integer values in ascending order.

#### Correlation analysis

**Figure 3:**
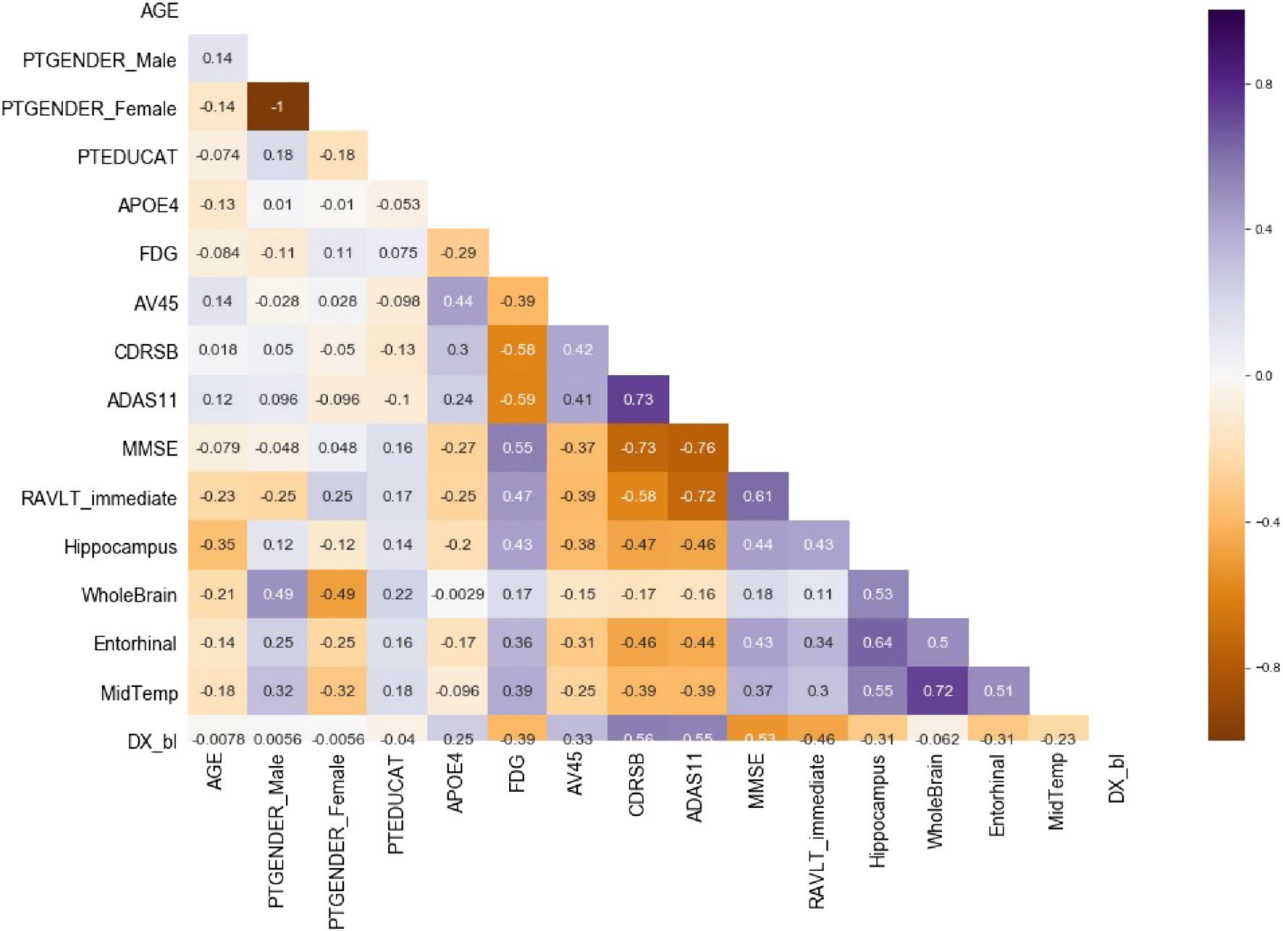
Linear correlation heat map for the data set.

Assuming correlation between two features, it means that one of them does not contribute to better representation of the information for the model to be learned, thus it can be omitted. Heat map representation as shown on Fig. 3 has been used to represent the linear correlations between the features in the data set used for this research.

The map provides a strong negative correlation of -1 between PTGENDER_Male and PTGENDER_Female. This coefficient does not provide anything relevant since both features are discrete and represent same category of data, so it will be ignored.

**Figure 4:**
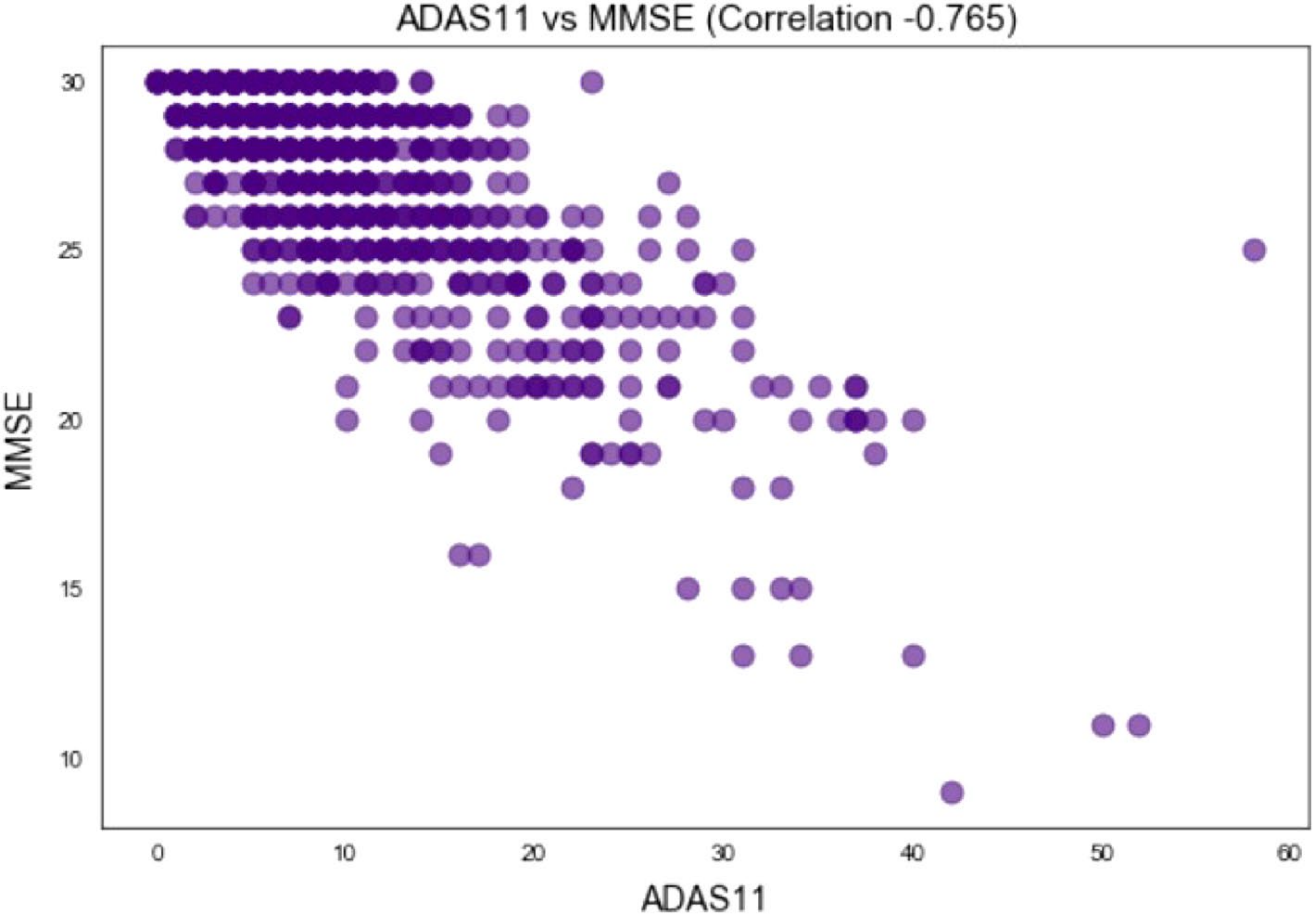
Scatter plot between ADAS11 & MMSE.

On the other hand, a trend of pretty high coefficients can be noticed between ADAS11 and other cognitive tests results. In fact, highest negative coefficient is between ADAS11 and MMSE (-0.76) and highest positive coefficient is between ADAS11 and CDRSB (0.73). It is possible that this feature does not provide any new information. It seems like it contains repetitive information from other tests. To determine the correlation, the coefficient alone is not sufficient. Additionally, graph-based representation (Fig. 4) is used between the two features to better understand the dependency.

From the plot a kind of linear dependency between these two features can be perceived. Plots of ADAS11 and CDRSB / RAVLT_immediate show something similar too. Considering the analysis, we assume that ADAS11 does not provide any new information, thus this feature is redundant and can be omitted from the final data set.

#### Missing values imputation

Discarding entire rows that contain missing values comes at the price of losing data which may be valuable, even though it is incomplete. In order to obtain the maximum information that the data set is providing, a missing values imputation is performed. Each imputation algorithm uses some kind of estimation to obtain missing values, but that does not mean that the value is 100% estimated correctly. This can cause noise and bias problems in the data set and that is why it should be used with caution.

One type of imputation algorithm is univariate, which imputes values in the $i-th$ feature dimension using only non-missing values in that feature dimension. Missing values can be imputed with a provided constant value, or using the statistics (mean, median or most frequent) of the particular column. This technique is also referred as simple imputing. By contrast, multivariate imputation algorithms use the entire set of available feature dimensions to estimate the missing values. These algorithms model each feature with missing values as a function of other features, and use that estimate for imputation. This technique is also referred as iterative imputing.

The goal is to compare different estimators to see which one is most effective for the data set. First, a subset of all rows with non missing value was extracted and its score was estimated. After that, a single value was randomly removed from each row and after iterative imputation of the missing values using different estimators, a score was estimated for each one of them.

#### Imbalanced targets distribution

Table 2 presents the targets distribution before and after elimination of subjects with lack of data, i.e. the distribution of the five different classes considering the data set in different stages.

**Table 2:** Original versus reduced targets’ distribution.

Diagnosis	DX_bl	Values	
		Original	Reduced
LMCI	3.0	4644	3526
CN	1.0	3821	2652
EMCI	2.0	2319	1854
AD	5.0	1568	1196
SMC	4.0	389	364

It can be noticed that there is a huge disproportion between the class with most values (LMCI) and class with least values (SMC). The aim is to create approximate uniform distribution of targets, such that each class will have similar number of instances. An undersampling of LMCI targets will be performed, combined with oversampling of other four classes.

Because the sampling processes mix original samples and artificially created ones, in order to be sure that the data set is not biased, first the data set is split into training (70%) and testing (30%) subsets and then separate sampling is performed into each one of them. Couple of different oversampling algorithms were tested combined with Random undersampling. The only exception is SMOTETomek algorithm which already combines methods for both oversampling and undersampling.

**Figure 5:**
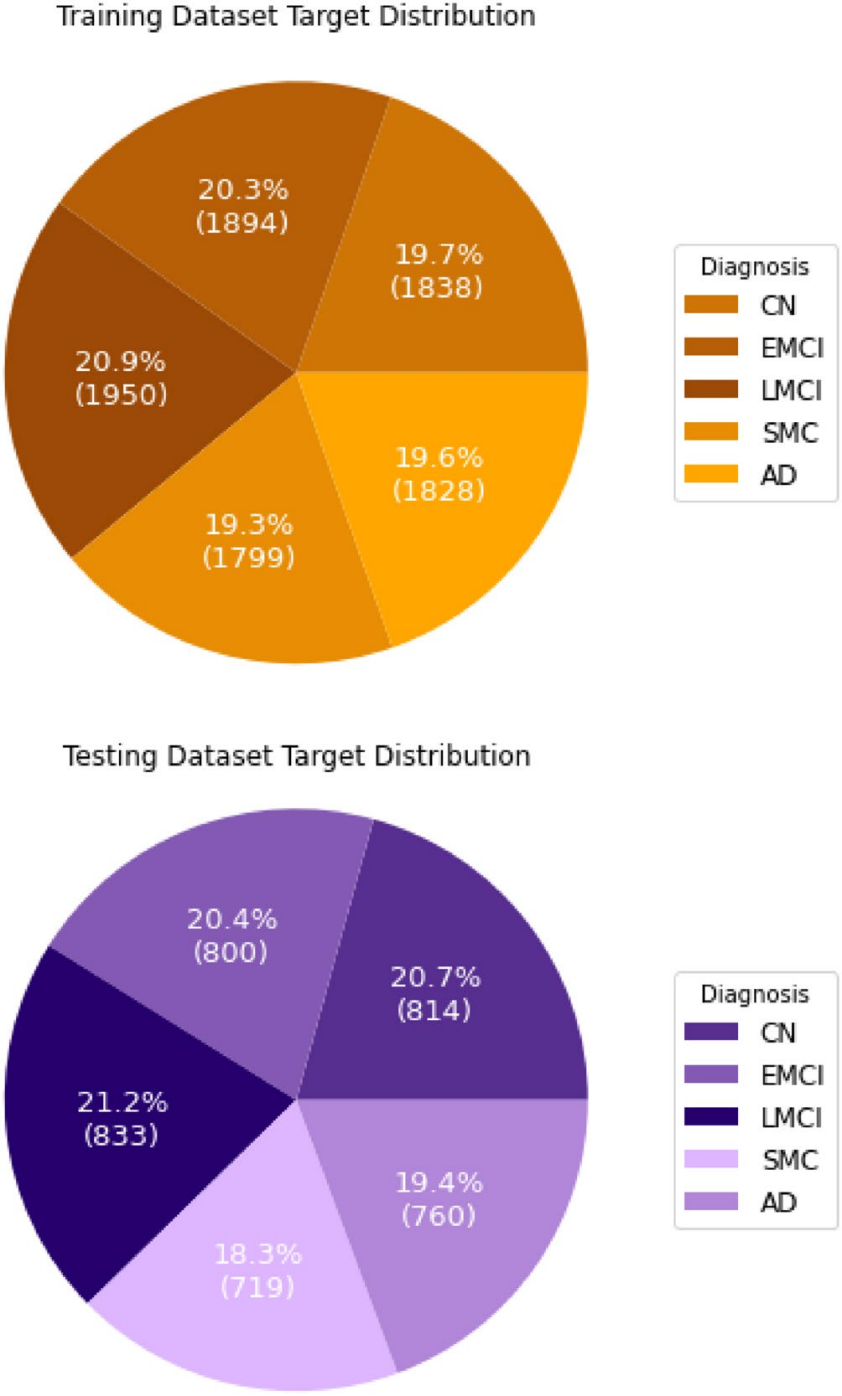
Targets’ distributions after data balancing.

The pie charts in Fig. 5 represent the number of targets divided by classes in both training and testing subsets after the sampling techniques were applied. It can be seen that they are now almost uniformly distributed.

### Machine Learning

Choosing the most optimal algorithm for solving the problem at hand depends on many factors like: size of the training data, training time, linearity, and number of features. The data set used is medium sized with average number of features allowing to experiment with more complex algorithms. Hence, the classifier built in this research uses **XGBoost** algorithm. Proven to show several advantages above other classification algorithms^47^, XGBoost requires less feature engineering, meaning there is no need for scaling and normalizing data. It is less prone to overfitting if the hyperparameters are tuned properly. For comparison purposes only, a Random Forest model was also built. To validate the trained classifiers properly, 5-fold cross-validation is performed on the training set.

One of the crucial steps in building ML model is tuning its hyperparameters - the arguments that can be set before training and which define how the training is done. These parameters are tunable and can directly affect how well a model trains. Thus, in order to achieve maximal performance, it is important to understand how to optimize them. To find the best combination of values for hyperparameters for both the Random Forest and XGBoost model, a range of values for every parameter has been defined and then Grid Search has been used which evaluates all combinations and chooses the best one.

### Model evaluation

Different performance metrics are used to evaluate the intelligent model built as described in the previous section. Balanced accuracy is considered a good measure for this research since it is reliable metric even when the distribution of target variable classes in the data is not very balanced. Precision is also considered informative measure since it tells the proportion of subjects diagnosed with one of the diagnosis (predicted class), that actually had that diagnosis (real class). Recall is used to measure the proportion of subjects that actually had particular diagnosis (real class) was diagnosed by the model to have that diagnosis (predicted class). Specificity is also used to measure the model’s ability to correctly generate a negative result for subjects who do not have the condition that is being tested. A high-specificity model will correctly rule out almost everyone who does not have the disease and will not generate many false-positive results. A model with high sensitivity but low specificity, results in many individuals who are disease free being told of the possibility that they have the disease, and are then subject to further investigation. The last metric used is F1-score, presenting the harmonic mean of both the precision and recall, and thus is considered to be very powerful for the problem at hand.

### Explainable machine learning

In the context of ML systems, interpretability is the ability to explain the model’s output. When a model is built, one need to be able to understand how it is making the predictions. Feature importance helps in estimating how much each feature of the data contributed to the model’s prediction. In this research the feature importance is considered in terms of **Shapley values** by using the SHAP (SHapely Addictive exPlanations) method^48,49^.

SHAP provides two aspects of model’s interpretability:

1. The first one is **global interpretability**-the collective SHAP values can show how much each predictor contributes, either positively or negatively, to the target variable. The summary variables importance plot shows the average impact each feature has for predicting each diagnosis, regardless if it is positive or negative. On regular variable importance plots, subjects are shown as colored dots. For each diagnosis there is a separate plot and for each feature, dots are arranged depending on the impact the value for that feature had on the subject to be predicted with the chosen diagnosis. The color of the dot indicates the value of the feature. For the dependence plots, dots are arranged on the x-axis by the value of the main chosen feature and on the y-axis by the positive/negative impact that value had for predicting the particular diagnosis. The color of a dot indicates the subject’s value for the second feature that the first one is in strongest interaction with.
2. The second aspect is **local interpretability**-each observation gets its own set of SHAP values. This greatly increases its transparency. These plots are subject specific. On the plots, blue arrows represent features that are increasing the predicting probability of a particular class (pushing it to the left), while red arrows are features that are decreasing the probability of a diagnosis to be predicted (pushing it to the right). Arrow’s length indicates how high the value of the feature is.

### Ethics approval and consent to participate

As per ADNI protocols, all procedures performed in studies involving human participants were in accordance with the ethical standards of the institutional and/or national research committee and with the 1964 Helsinki declaration and its later amendments or comparable ethical standards. More details can be found at adni.loni.usc.edu. This article does not contain any studies with human participants nor animals performed by any of the authors.

### Consent for publication

Authors proclaim that all terms of the data use agreement are accepted and included in the manuscript. The manuscript has been sent to ADNI Data and Publication Committee and it has been approved for publishing. Authors acknowledge that all images are entirely unidentifiable and there are no details on individuals reported within the manuscript.

## Results

### Data preprocessing

**Figure 6:**
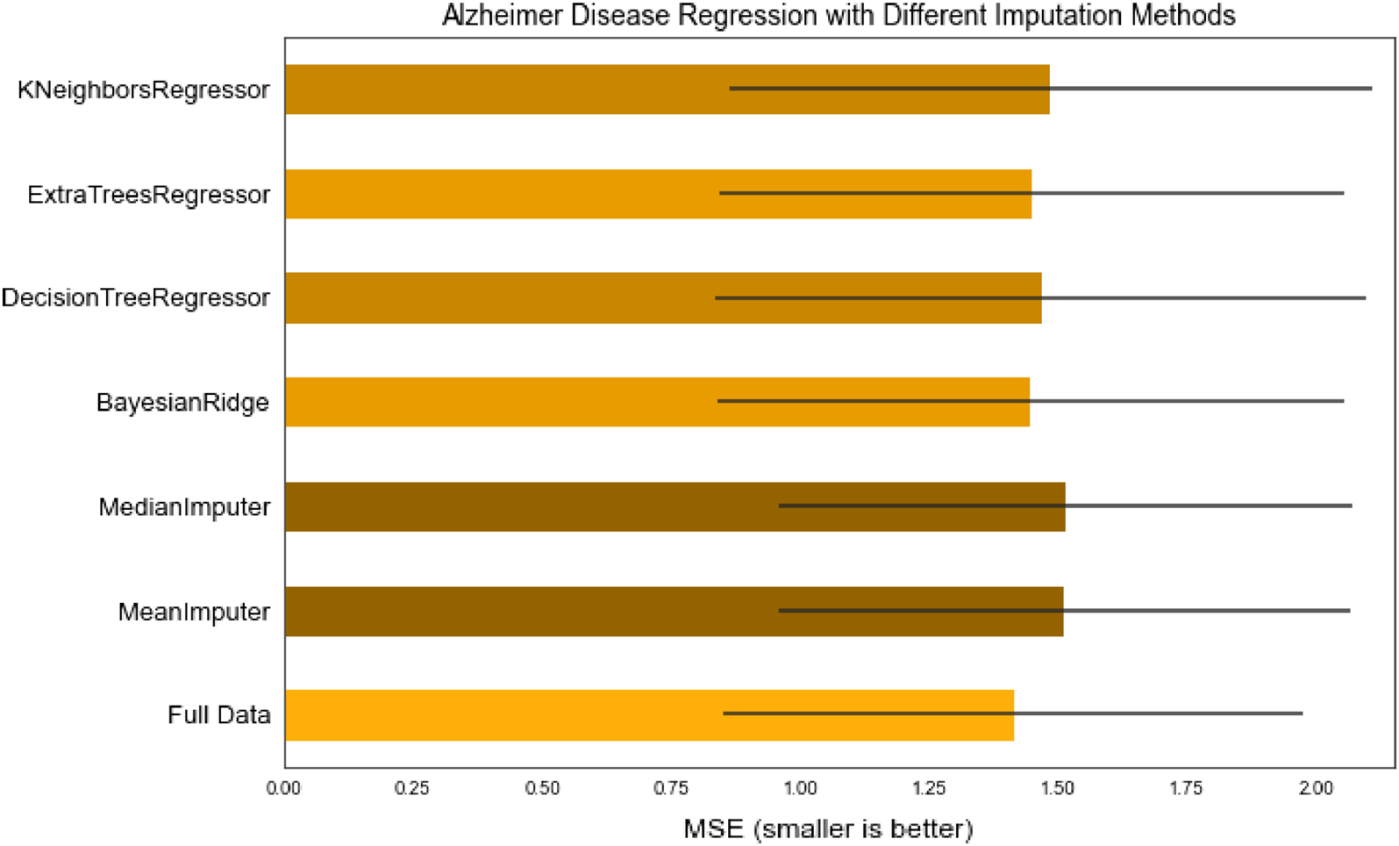
Comparison of multiple imputation algorithms performance.

Considering the data imputation methods applied, as it can be seen from the bar chart presented in Fig. 6, Extra Trees Regressor and Bayesian Ridge estimated values are closest to the original data. Both of them are multivariate algorithms. On the other hand, univariate algorithms using mean and median failed to do the estimation very accurately, which is somewhat expected considering the fact that the data set contains features represented by sensitive data values where simple average does not solve the problem.

In addition, k-Nearest Neighbors imputation approach was performed. By default, a euclidean distance metric that supports missing values is used to find the nearest neighbors. Each missing feature is imputed using values from k-nearest neighbors that have a value for the feature. Although this type of imputation showed better results than the simple imputation algorithms, it did not surpass the iterative imputation using Extra Trees Regressor.

Considering the results from the comparison, the Extra Trees Regressor was chosen to impute the missing values in the original data set. After that, several such regressors with different number of estimators were tested on the original data set. Experiments have shown that Extra Trees Regressor with 100 estimators most effectively approximates the missing values.

**Figure 7:**
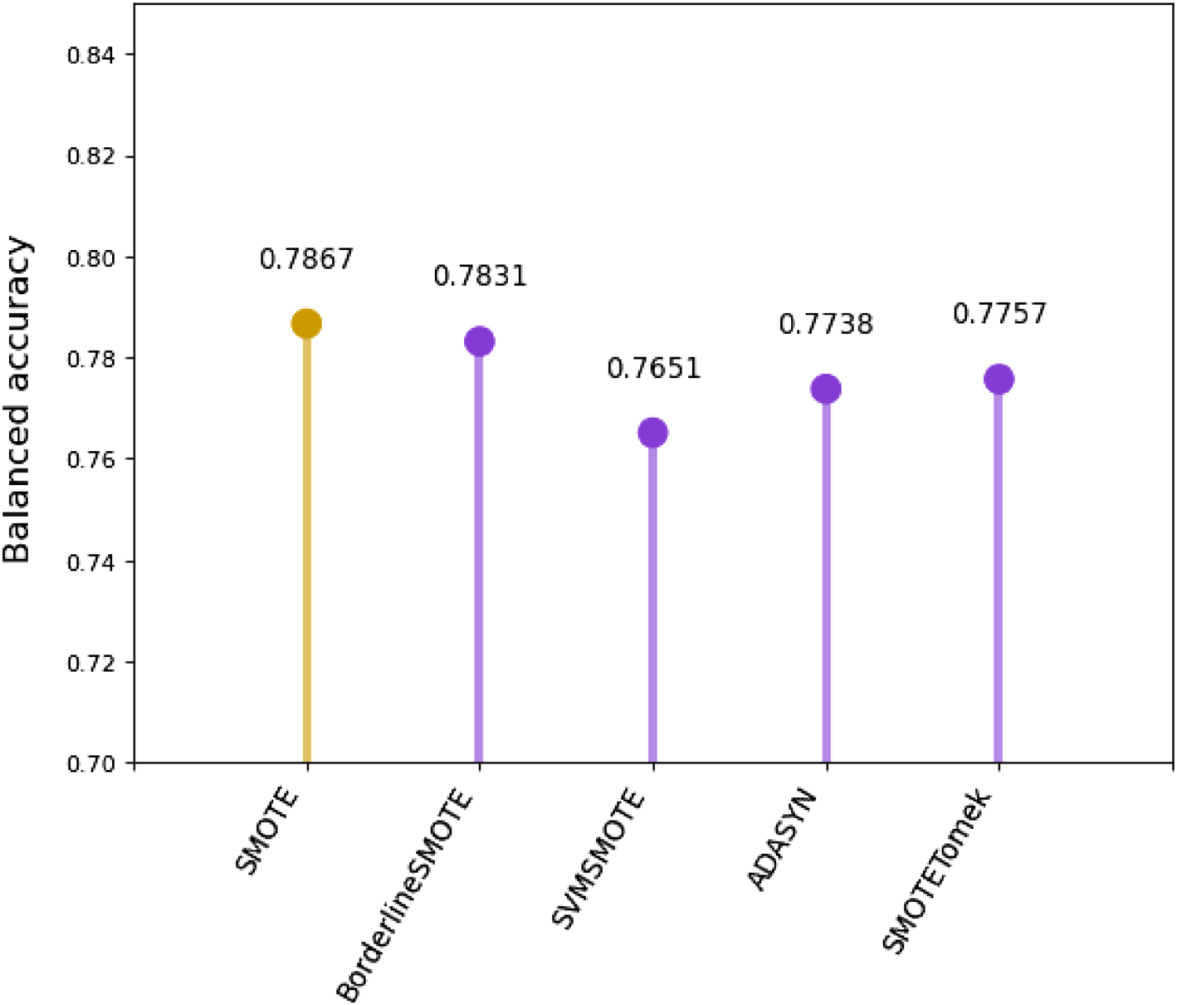
Oversampling algorithms comparison.

**Figure 8:**
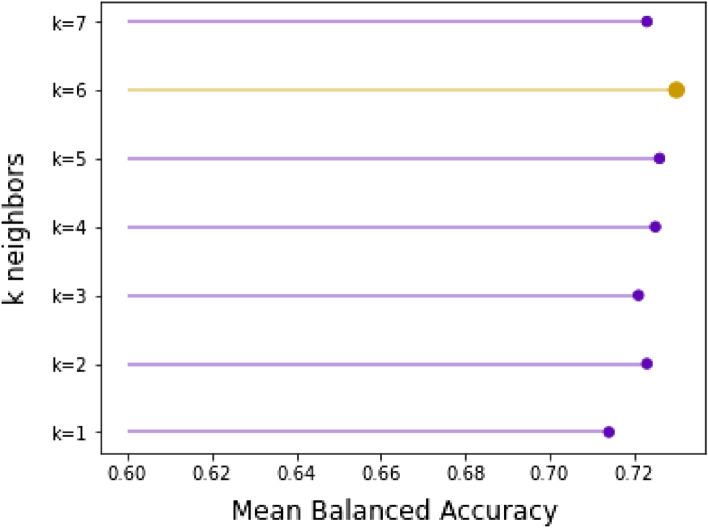
K-neighbours validation.

Evaluation results suggest that the SMOTE oversampling algorithm provides the best results as shown in Fig. 7, although the difference from one to another is very minor. Another area to explore would be to test different values of the k-nearest neighbors selected in the SMOTE procedure when each new synthetic sample is created. The default is k=5, although larger or smaller values will influence the types of samples created, and in turn, may impact the performance of the model. The repeated and stratified k-fold evaluation showed that the oversampling process is most successful when the number of k-nearest neighbors is equal to 6 as depicted in Fig. 8.

Completing this step, the data preprocessing phase is finished and the data set is ready to serve as a source for building the intelligent models. The resulting data set after the preprocessing consists of 13,235 subjects.

### XGBoost model evaluation

**Figure 9:**
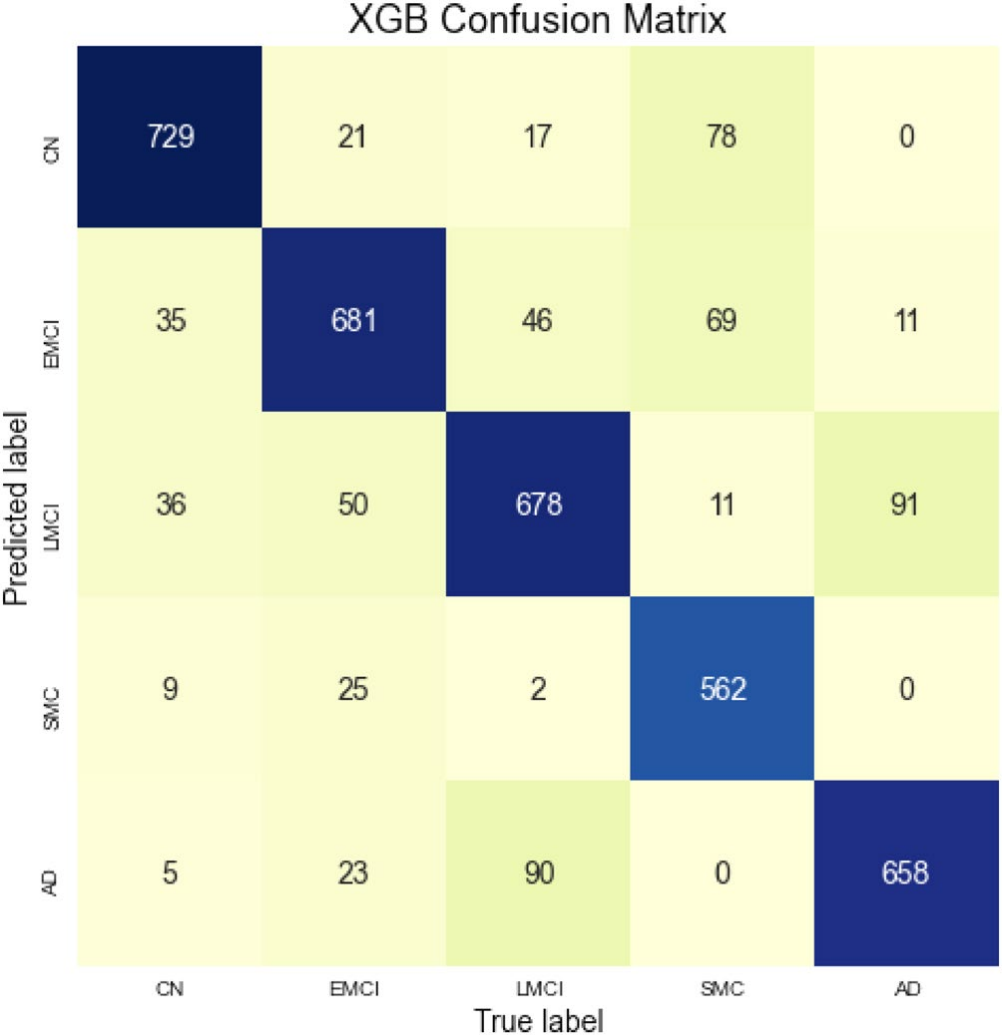
Confusion matrix for the XGBoost model.

The confusion matrix for the XGBoost model is presented on Fig. 9. Majority of subjects lay on the main diagonal. One of the model’s anomalies is the slightly increased number of AD patients predicted with a LMCI diagnosis. The same problem occurs with LMCI patients that are predicted with an AD diagnosis. This tells that perhaps there are some features that bring confusion between these two diagnoses. In addition, there is an increased trend into false negatives of the SMC class, but considering the fact that serious amount of the SMC subjects were created artificially, this phenomenon is somewhat understandable.

Table 3 shows complete evaluation of XGBoost performance, divided by target classes. XGBoost model managed to achieve balanced accuracy of 0.84. Other metrics are also very satisfactory, deviating from 0.8 to 1.0. Table 4 shows comparison between this model’s metrics and results obtained using Random Forest model. XGBoost managed to guess more subjects with their correct diagnosis, thus it represents more accurate model.

**Table 3:** XGBoost classification performance for each class.

	Precision	Recall	Specificity	F1-score
CN	0.86	0.90	0.96	0.88
EMCI	0.81	0.85	0.95	0.83
LMCI	0.78	0.81	0.94	0.80
SMC	0.94	0.78	0.99	0.85
AD	0.85	0.87	0.96	0.86

**Table 4:** Comparison of models’ performance.

	Precision	Recall	Specificity	F1-score	Accuracy	Training time	Prediction time
XGBoost Classifier	0.85	0.79	0.96	0.84	0.842	20.8 s	103 ms
Random Forest Classifier	0.78	0.79	0.95	0.79	0.787	5.16 s	157 ms

### XGBoost model interpretability

#### Global interpretability

**Figure 10:**
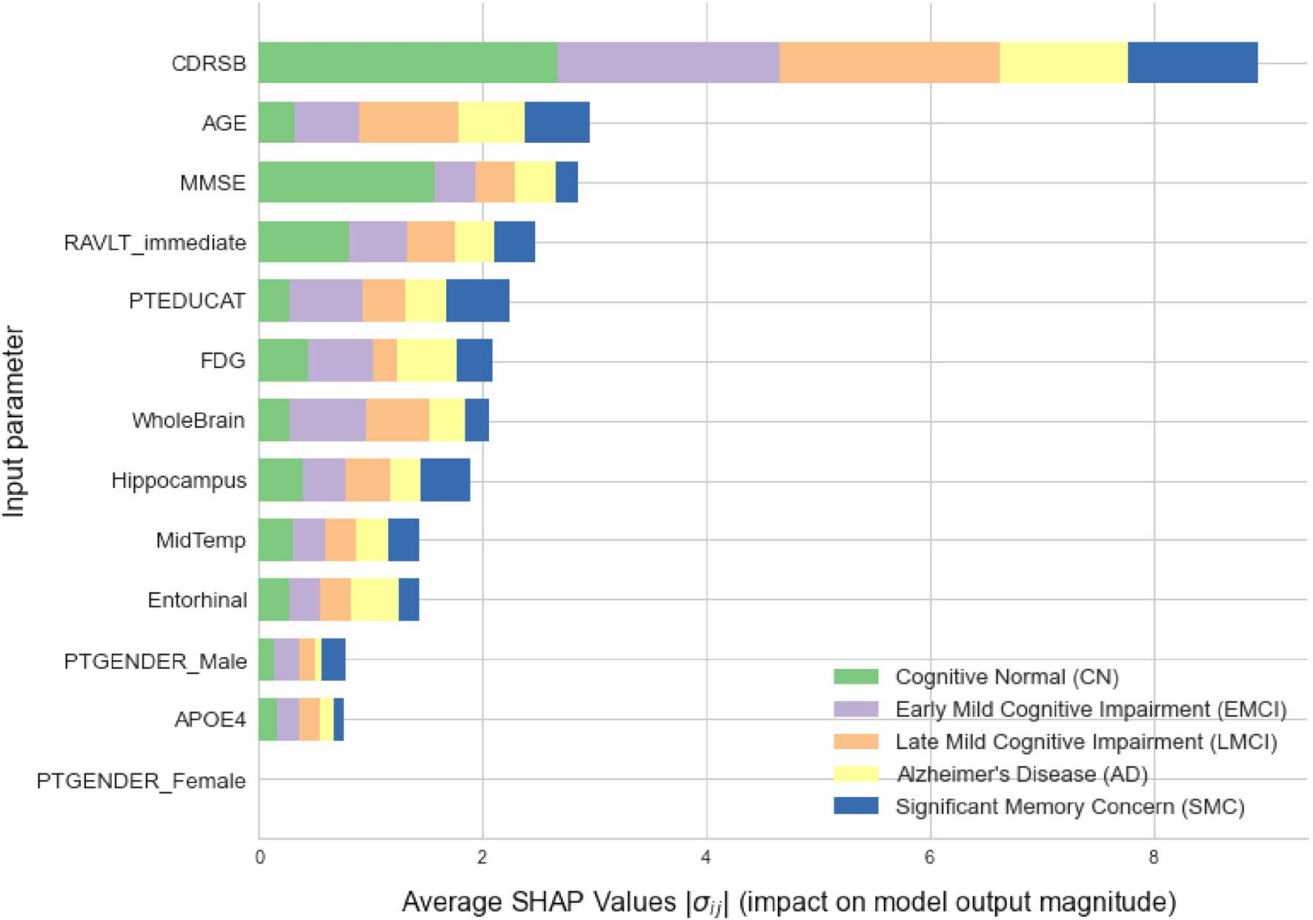
Summary variables importance plot for XGBoost model.

The following Fig. 10 presents how high is each feature influence (variables importance) on each group of targets in average. Analysing the plot, it can be noticed that CDRSB leads by far the most in the impact on this model’s output. All features that describe cognitive tests results are generally ranked on the top. Another thing worth mentioning is the position of FDG and APOE4. Since PTGENDER_Male and PTGENDER_Female are highly correlated and basically represent one feature, impact values of PTGENDER_Female are omitted here. Note that the gender and APOE4 features are at the very bottom, which means their values influence the outcome least. This information tells us that probably there is not a gender predisposition for obtaining Alzheimer’s disease. Many studies^50,51^ say that e4 alleles of the APOE gene can be a predisposition for dementia, but not necessarily. From this result, it can be confirmed that the APOE gene does not act as a decisive factor for having a diagnosis.

Looking at the color distributions at some features, it can be seen that MMSE value impacts most on the CN subjects. On the other hand, subject’s age has most influence for LMCI class. For the AD class, the impact of the gender feature is insignificant. If we take a closer look on the green parts of each feature, we can notice that cognitive tests have highest impact on the CN class, while other features impact the output for this class in smaller amount. This analysis is done on a basis of complete training dataset.

**Figure 11:**
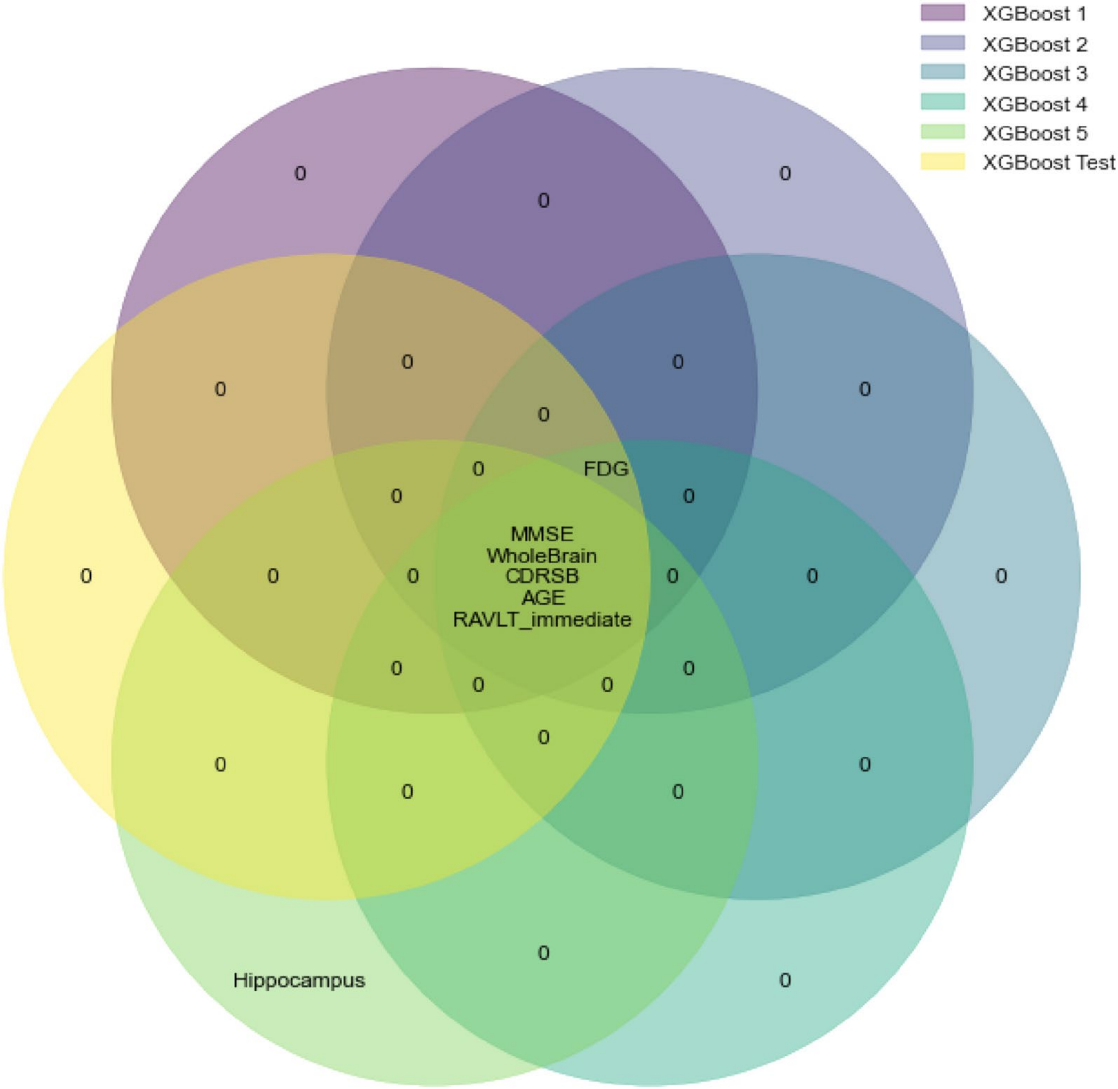
Venn diagram presenting features impact ranking for various XGBoost models based on 5-fold cross-validation.

Of significant importance is to prove robustness of the present conclusions, following the idea that the order of features importance is not linked only to the particular observation. To accomplish this, the training dataset was split using stratified 5-folds cross-validation technique. For each split, a new model with the same properties as the original one was built and trained on 4/5 folds of data. For each of these models, SHAP values of their training subsets were obtained indicating their most important features in descending order. Additionally, most important features based on the original testing dataset were obtained from the original model. The following approach provides information in which manner models using different datasets have learnt and ranked features importance compared to the ranking of features importance based on subset of data not recognizable for the model. Top 6 most influential features were selected for each model and using a Venn diagram visualization, their overlaps and intersections are presented in Fig. 11. Circles for XGBoost 1 to XGBoost 5 represent features importance originating from 5-fold cross-validation while XGBoost Test circle represents features obtained from the original testing dataset. On the diagram, it can be noticed that 5 out of 6 top features overlap in all six models. The only mismatch is occurring at model XGBoost 5 where instead of FDG, the feature Hippocampus is included. But, if we take the top 7 features, this will be over-passed since the 7th feature in the list for XGBoost 5 is indeed FDG. Observing the bigger picture, differences between a feature ranking in all models are happening only for a place or two. There are no large mismatches between the rankings, indicating that the global interpretability presented before is pretty stable and robust even for models using to some extent different datasets. In addition, the robustness of the interpretability method is extended over different tree-based algorithms too, indicating the independence between the selected algorithm and features influence on predicting the target variable. The discussion in Appendix A.2.1 provides evidence on obtaining the same top features for the random forest model in comparison to the XGBoost.

**Figure 12:**
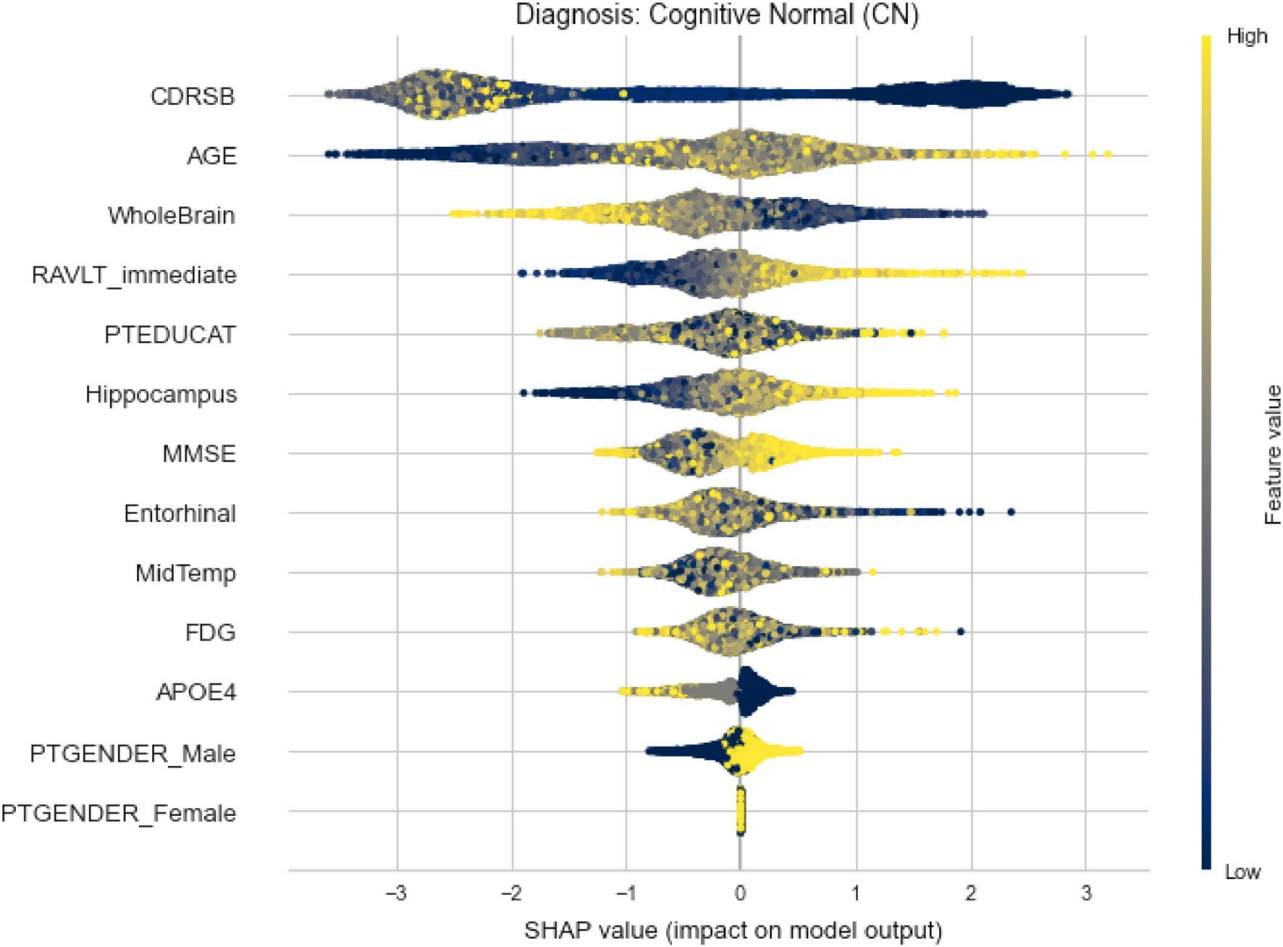
Variables importance plot for CN diagnosis.

**Figure 13:**
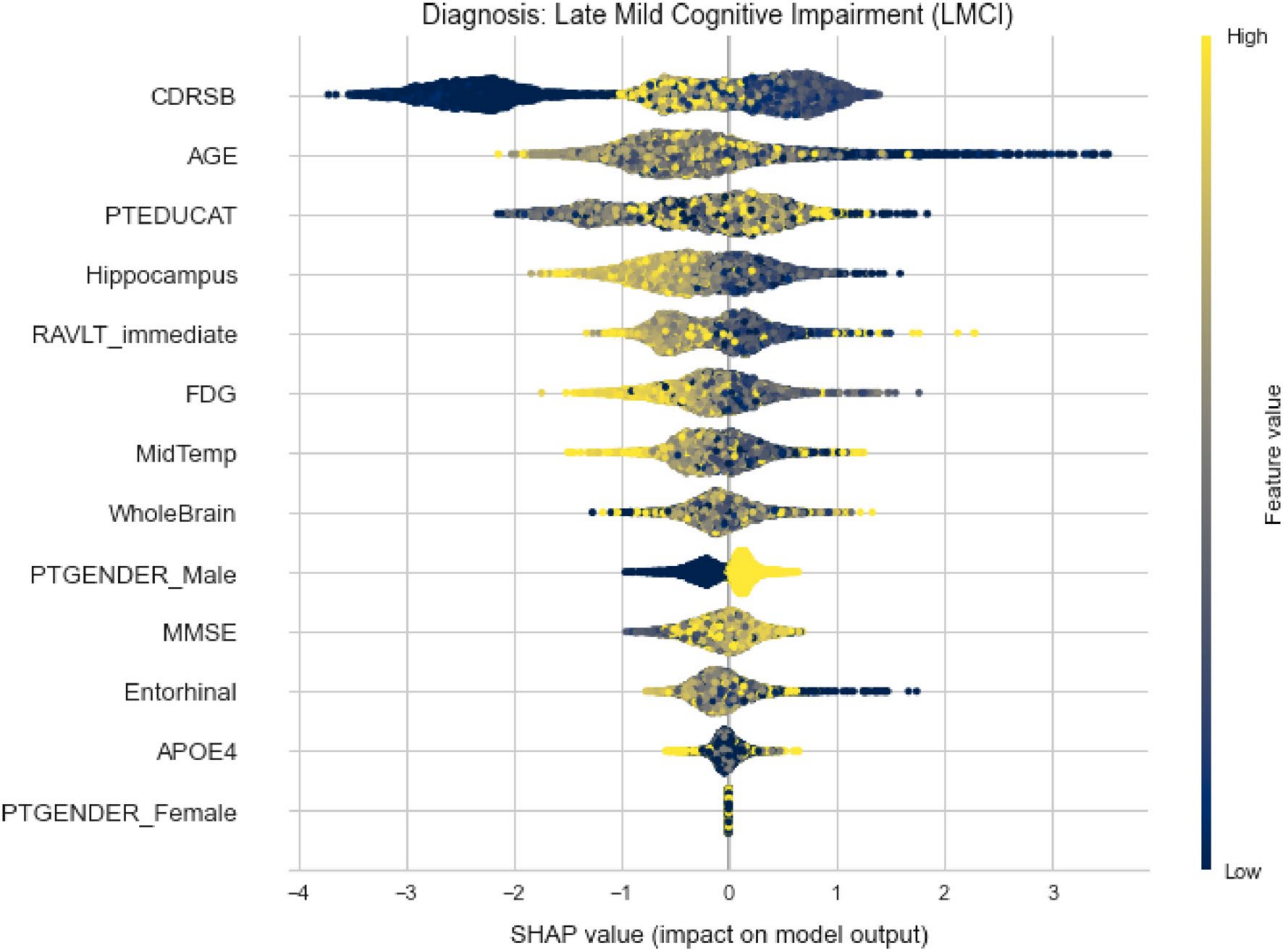
Variables importance plot for LMCI diagnosis.

Considering the global interpretability, we can also take a closer look of whether one feature has positive or negative impact on the output and how high is it really, by looking at the summary plot for each diagnosis. The plot is made of all the dots in the train data. Figures 12 and 13 depict the summary plots of variables importance for CN and LMCI class diagnosis.

On the summary plot of CN class, we can notice that low values of CDRSB have very high positive impact determining this diagnosis. Additionally, high values of MMSE and RAVLT_immediate also tend to have high positive impact. While other MRI measures do not give useful information on how they affect the model’s output, the WholeBrain feature have serious negative impact for higher values and vice versa. The same statement applies to Hippocampus too, but not to that extent.

Contrary to already mentioned conclusions, it can be noticed that younger subjects tend to decline from this diagnosis. Although the natural way of thinking is that neurological diseases affect only older people, that is not the truth. In addition, from TADPOLE data set it can be concluded that women are more inclined to neurodegeneration.

For the LMCI class, shown in Fig. 13, the situation is slightly different, as expected. Low AGE values are distributed on the positive axis now, denoting positive impact for this class. It can be seen that lower values for CDRSB have highly negative impact on this class, high values do not have any impact at all, while mid values have positive impact. It is very likely that high values are going to be distributed on the positive axis for the AD class. Here, high values of RAVLT_immediate have negative impact, which is somewhat expected. Other features (Hippocampus, FDG and MidTemp) have similar types of distributions denoting negative impact on model’s output for higher feature’s values.

**Figure 14:**
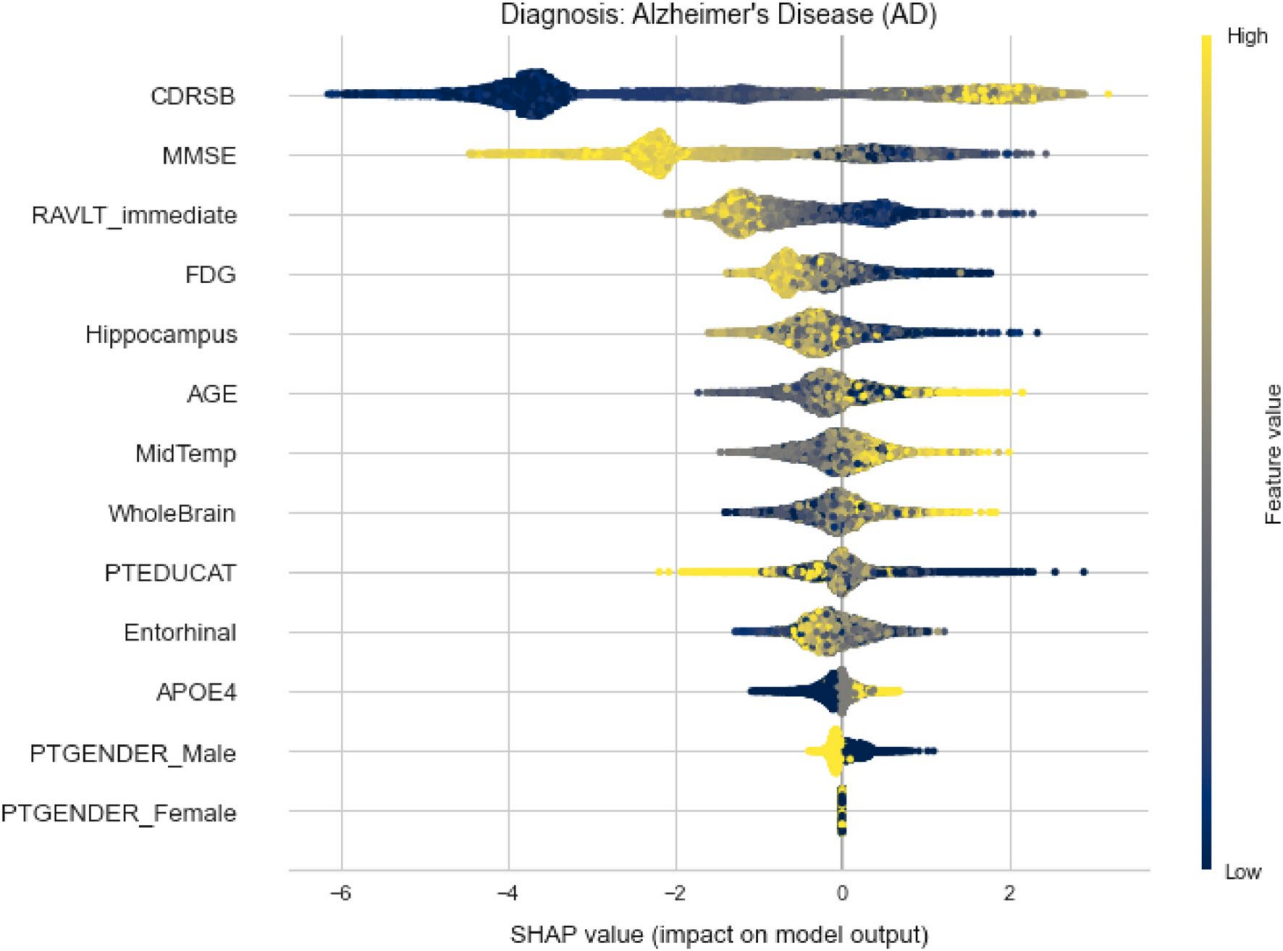
Variables importance plot for AD diagnosis.

Figure 14 shows that high CDRSB values indeed represent high positive impact for AD class. In opposite, higher MMSE values have significant negative impact. Looking at the PTEDUCAT feature, it can be seen that higher education has valuable negative impact on the Alzheimer’s diagnosis. Also, the APOE4 distribution indicates that subjects with zero e4 alleles have less chance of being labeled with AD than those with one or two. It can also be confirmed that subjects with lower values of FDG tend to be diagnosed with this diagnosis.

Even more information can be uncovered examining the partial dependence plot of one feature. This plot shows the marginal effect two features have on the predicted outcome. Once the first feature is chosen, the second is automatically selected depending with which one the first interacts most. These plots are also class specific.

Most of the plots show more complex correlation, or no correlation at all, but those that are most relevant will be examined.

**Figure 15:**
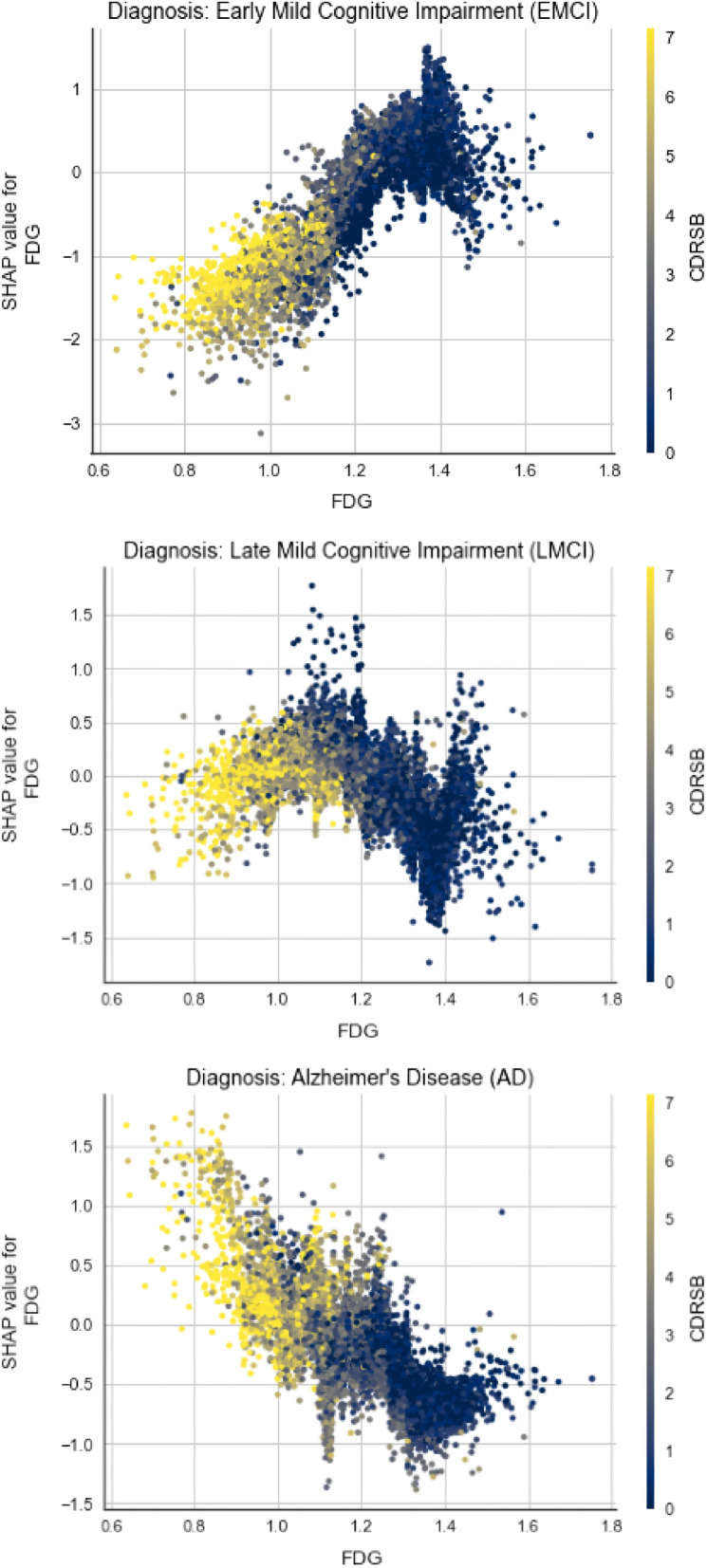
Dependence plots between FDG and CDRSB for different classes.

First, the marginal effect FDG and CDRSB have on the predicted outcome is analysed for three different classes (EMCI, LMCI, AD) on Fig. 15. On all three plots we can notice the correlation between FDG and CDRSB, as subjects with lower FDG tend to have higher CDRSB results. The plot color changes progressively from yellow to blue, as we move on the x-axis from 0.6 to 1.8. But what fascinates the most is how the positive correlation that they have with the outcome gradually evolves into negative one, as the disease stages move from cognitive normal to Alzheimer’s disease.

**Figure 16:**
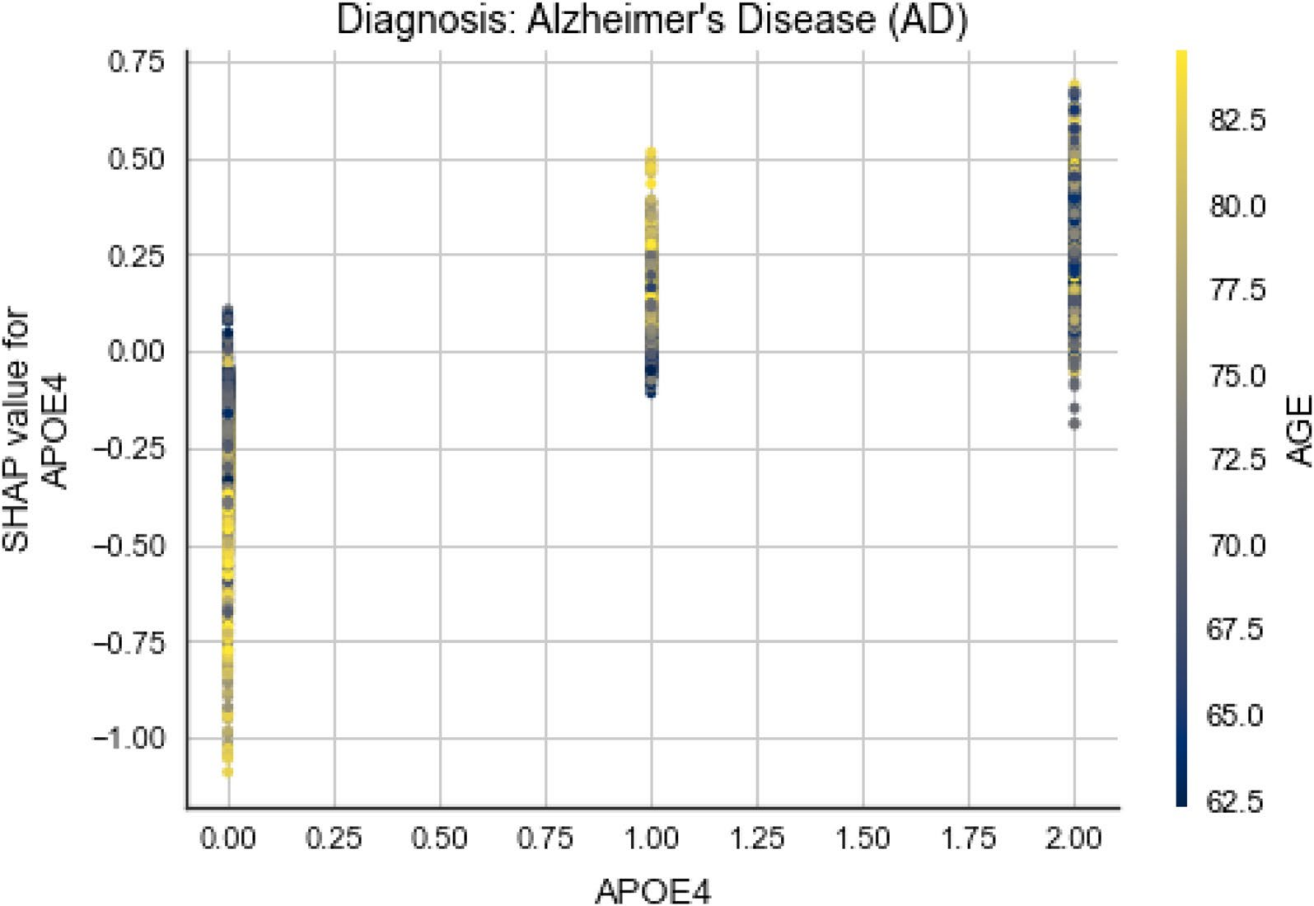
Dependence plot between APOE4 and AGE for AD class.

In Fig. 16, it can be seen that younger subjects with two alleles e4 of APOE gene have bigger chances of having an Alzheimer’s disease. This represents intriguing information about the genetic predisposition of the disease, since we can see that APOE4 and AGE are correlated somehow. In addition, high values for AGE and zero e4 alleles have negative impact on this class. Contrary, one e4 allele and higher age values have a positive impact.

**Figure 17:**
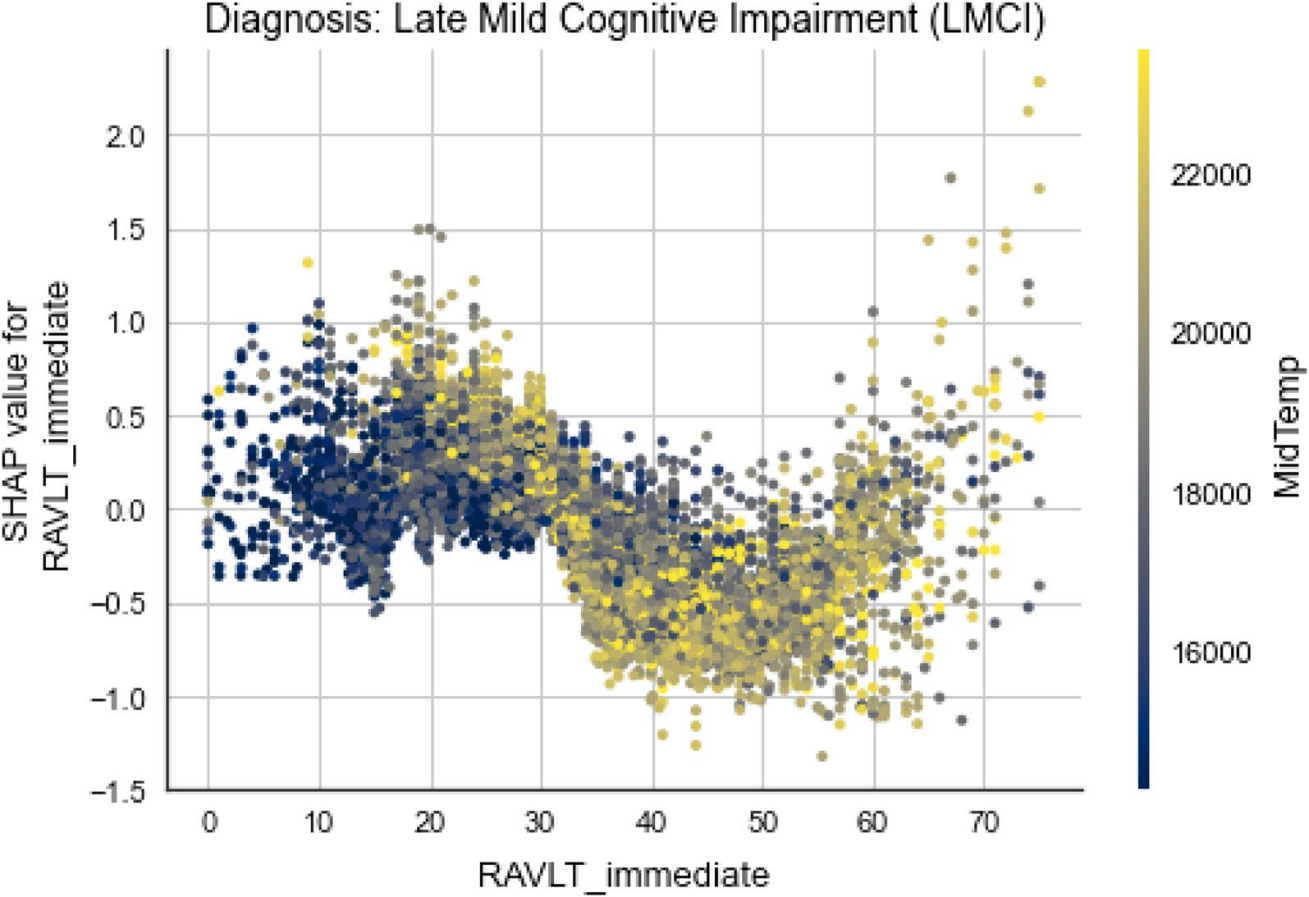
Dependence plot between RAVLT_immediate and MidTemp for LMCI class.

Last but not least, there is pretty complex correlation between RAVLT_immediate and MidTemp and they also influence the output in non-straightforward way, thus a couple of statements can be made from Fig. 17. Diving deeper into the plot, it can be seen that subjects with lower values for RAVLT_immediate tend to have lower values for MidTemp too and they have mild positive influence for LMCI class. On the other hand, higher values for the cognitive test correlated with mid to high MidTemp values tend to have negative impact.

#### Local interpretability

**Figure 18:**
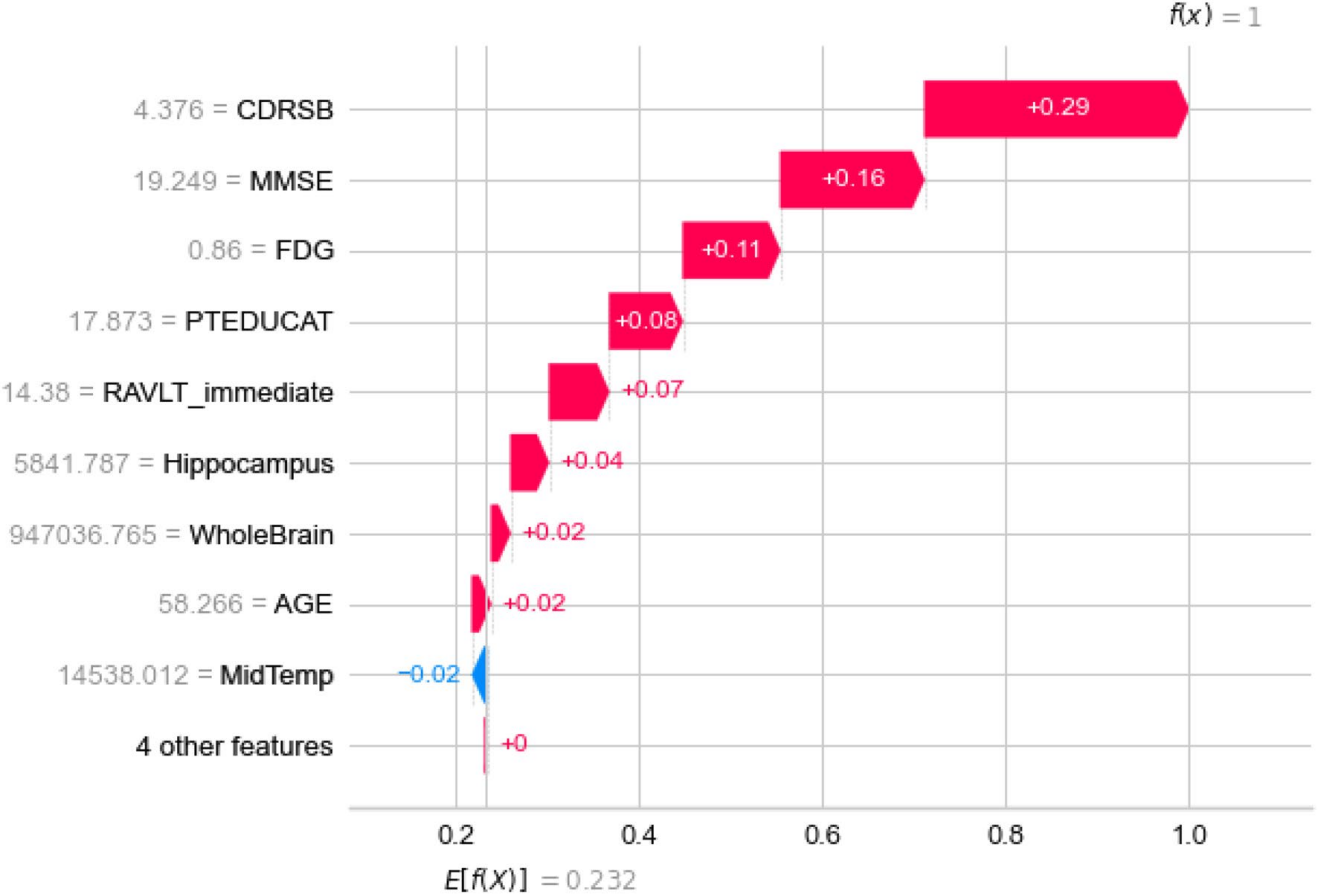
Features influence on a subject with AD diagnosis to be predicted as AD.

**Figure 19:**
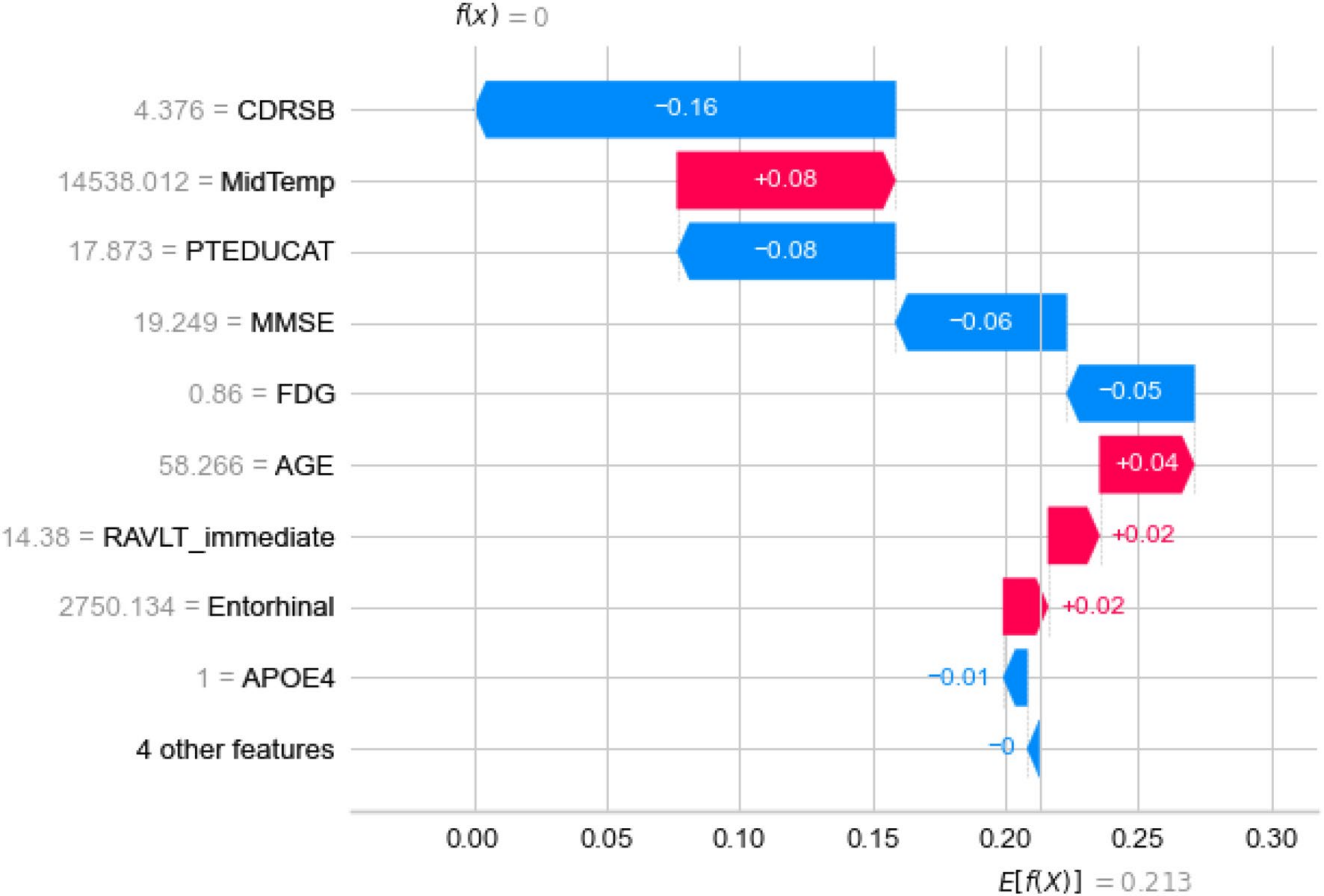
Features influence on a subject with AD diagnosis to be predicted as LMCI.

By isolating a single subject, it can be explained graphically how features influence the subject to become labeled with particular class. One subject belongs to all classes with different probabilities and is labeled with the class with highest probability. Let’s consider a subject that was correctly predicted with AD diagnosis as shown in Fig. 18. The graph presents an **output value** or **f(x)** which is prediction probability for the particular observation, and **base value** or **E[f(x)]** which is the value that would be predicted if any features are not known for the current output (*mean prediction*). Observing the waterfall plot in Fig. 18 for the correctly predicted class and the LMCI class in Fig. 19, the following conclusions can be derived.

As it is expected for the AD class, almost all features have positive impact, except the MidTemp with significant weak negative impact. The prediction is straightforward with pretty high probability. On the other side, the prediction for the LMCI class for the same subject have pretty mixed up feature impacts. We can see that some of the features influence positively in the favor of LMCI class, but other features have higher negative impacts together, thus the subject’s probability to belong to this class is pretty low. In both cases, the CDRSB impact is crucial.

**Figure 20:**
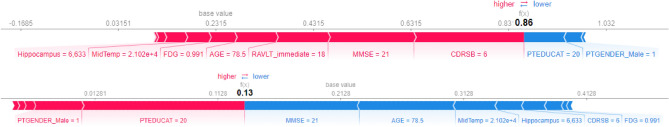
Comparison between features impact on the predicted (AD) and true class (LMCI) of a subject.

**Figure 21:**
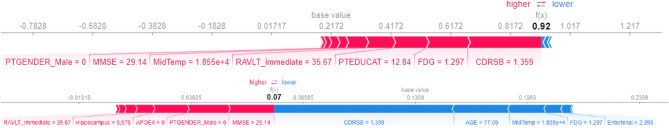
Comparison between features impact on the predicted (EMCI) and true class (SMC) of a subject.

**Figure 22:**
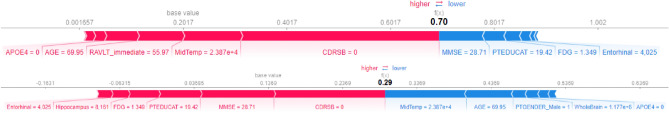
Comparison between features impact on the predicted (CN) and true class (SMC) of a subject.

**Figure 23:**
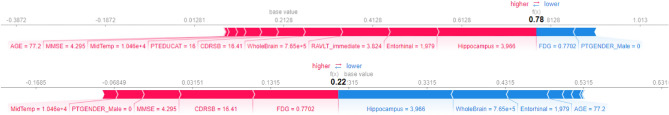
Comparison between features impact on the predicted (LMCI) and true class (AD) of a subject.

This interpretation can be used to analyse the trends of false predicted subjects mentioned before. The features influence is examined on the predicted and true class of randomly selected subject belonging to the particular trend. For each of the Figs. 20, 21, 22, and 23 top plot represents the predicted class, while the bottom plot is for the true class.

On Fig. 20, a subject with **LMCI diagnosis, predicted as AD** is shown. It can be noticed that the difference between probability for the predicted class and the true one is slightly big. Besides CDRSB, in this case MMSE and AGE play significant role in increasing one’s probability and decreasing the other. It seems like most of the patient’s medical measurements indicate an AD instead of LMCI.

Figure 21 shows individual plots of a subject with **SMC diagnosis, predicted as EMCI**. Even bigger probability difference occurs here. On the first plot, almost all features influence positively in favor of EMCI diagnosis. The problem with many false predictions correlated to the SMC class may be because of the initially deficient subset of targets we were dealing with at the beginning. The low CDRSB value influences the most against the true class and in favor of the predicted one, which was indeed proven (higher CDRSB values tend to correlate with more devastating diagnoses).

In the next two Figs. 22 and 23, plots of a **subject with SMC diagnosis, predicted as CN** and **subject with AD diagnosis, predicted as LMCI** are shown. The probability difference in both cases is slightly smaller than before, but it is still pretty high. In the first case, CDRSB has positive impact for both classes, but for the predicted one it is slightly higher. MidTemp and AGE are playing the main role here in deciding the final output. In the last Fig. 23, MRI measurements have greatest impact on the subject. It can be seen that they have serious positive impact for the predicted class, in the same time decreasing the probability for the true class.

## Discussion

The results are summarized in the Table 4. For summarizing the categorical metrics, weighted average representation has been used. Although the main goal in this research was not achieving highest performances, it still can be confirmed that comparing to a Random forest model, the XGBoost algorithm performs better classification of subjects and better distinguishes between different types of neurodegenerative diagnoses, indicating that it is more reliable and accurate than his opponent in this research. Detailed explanation of Random forest model evaluation and interpretability can be found under Appendix A.

The global interpretability of the intelligent model provided exceptional conclusions that were deeply explained in the previous section. However, not all of the conclusions were comprehensively discussed and thus, the features influence on predicting a particular class has been summarized in Table 5. The table provides detailed scheme of the positive (+), negative (-), and undefined (X) influence the particular value of the feature has on predicting the particular class. This scheme can be used as additional knowledge for the physicians and other related experts when they are making conclusions over the diagnosis for particular patient.

**Table 5:** XGBoost global interpretability.

Diagnosis	Demographics						Cognitive scores						MRI								PET		Genotype		
	Gender		Age		Education		CDRSB		MMSE		RAVLT_immediate		WholeBrain		Hippocampus		Entorhinal		MidTemp		FDG		APOE4		
	M	F	Young	Old	Low	High	Low	High	Low	High	Low	High	Low	High	Low	High	Low	High	Low	High	Low	High	0	1	2
CN	+	−	−	X	X	+	+	−	−	+	−	+	+	−	−	+	+	−	−	X	+	X	+	−	−
EMCI	−	+	+	−	−	X	−	+	−	X	−	−	−	+	−	+	−	+	−	+	−	+	−	X	+
LMCI	+	−	+	X	X	X	−	−	−	X	+	−	X	X	+	− lePara>	+	X	+	−	+	−	−	X	X
SMC	−	+	−	−	−	+	+	−	−	X	−	+	−	+	−	+	−	−	−	−	−	+	X	X	−
AD	−	+	+	+	+	−	−	+	+	−	+	−	−	+	+	−	−	−	X	+	+	−	−	+	+

Considering the table of influences for the XGBoost model, it can be once again confirmed that XGBoost provides high interpretability of the problem, since there is very small amount of undefined influence statuses. Table 6 represents a merged table between the XGBoost’s and Random forest’s scheme. Luckily, there is a low level of inconsistency between the unveiled influence provided by both of the models. It is very rare that one model is contradictory to the other, e.g. the high education positive or negative influence on the SMC class is suspicious, and also APOE4 2 influence on the EMCI class is a matter of question whether it is positive or negative. The merged table provides achievement of a better understanding, unveiling the contribution to the undefined influence in each of them by masking two contradictory influence as undefined. Features influence for the Random forest model can be found in Supplementary Table S.2.

**Table 6:** Merged scheme showing the influence of each feature on each of the diagnosis.

Diagnosis	Demographics						Cognitive scores						MRI								PET		Genotype		
	Gender		Age		Education		CDRSB		MMSE		RAVLT_immediate		WholeBrain		Hippocampus		Entorhinal		MidTemp		FDG		APOE4		
	M	F	Young	Old	Low	High	Low	High	Low	High	Low	High	Low	High	Low	High	Low	High	Low	High	Low	High	0	1	2
CN	+	−	−	X	X	+	+	−	−	+	−	+	+	−	−	+	+	−	−	−	X	X	+	−	−
EMCI	−	+	+	−	−	X	−	+	−	+	−	−	−	+	−	+	−	+	−	+	−	+	−	+	X
LMCI	+	−	+	−	−	+	−	−	−	X	+	−	X	−	+	−	+	−	+	−	+	−	−	X	+
SMC	−	+	−	−	−	X	+	−	−	+	−	+	−	+	−	+	−	−	−	−	−	+	X	X	−
AD	−	+	+	+	+	−	−	+	+	−	+	−	−	+	+	−	−	−	X	+	+	−	−	+	+

Table 4 also shows the times required for the models to be trained and to predict the entire test set. The XGBoost classifier requires more time to be trained since it uses gradient boosting algorithms in background, but its predictions are slightly faster than the ones of Random Forest classifier. Benefits obtained in terms of model’s exactness and validity for the time difference are more than worthy.

It can be concluded that XGBoost is proven to be an optimal algorithm for dealing with Alzheimer’s prediction problem using the particular data set. Table 7 presents a comparison of our XGBoost model with other models trained on the same original TADPOLE data set^52^. As shown it can be perceived that our model is ranked second, and if a performance time is included as trade-off, then our model would be on the top of the list.

**Table 7:** Comparison of our model with some of the contestants of TADPOLE Challenge^52^.

	Feature selection	Features	Missing data imputation	Prediction model	BCA	Training time	Prediction time (per subject)
Frog	Automatic	490	None	Gradient boosting	0.849	1 h	–
Our XGB Model	Manual	13	Extra Trees Regressor	XGBoost	0.842	20.8 sec	0.03 ms
BenchmarkSVM	Manual	6	Mean of previous values	SVM	0.764	20 sec	0.001 sec
SMALLHEADS - NeuralNet	Automatic	376	Nearest neighbour	Deep NN	0.605	40 min	0.06 sec
Rocket	Manual	6	Median of diagnostic group	Linear mixed effects model	0.519	5 min	0.3 sec

Considering the popular AD research, mostly considered those published in the latest years, Table 8 presents an interesting review not only of the obtained results and the data set used, but also on the experimental setup performed in each of the reported experiments. High accuracy metrics are reported, however most of them show no clear evidence on appropriate methods for data preprocessing, missing data imputation, hyperparameters tuning, appropriate split of train and testing set, and the data sets used are limited to at most nearly two thousand patients. The most remarkable research among the presented is the one published in^53^ in which very comprehensive mathematically supported approach is presented with special attention on avoiding over/underfitting problems.

**Table 8:** Analysis of the latest eminent literature.

Reference	Dataset characteristics					Methodology	Results	Comment
	Size	Features	Origin	Description	Split			
^56^	1909 subjects (MCI or AD)	44	Coalition Against Major Diseases (CAMD)	ADAS-Cog and MMSE scores, laboratory and clinical tests, background information	Train: 75% Validate: 5% Test: 20%	Conditional Restricted Boltzmann Machine (CRBM)	Accuracy: 0.5 (differentiation between actual and synthetic patient data) R2: 0.820.01 (observed vs. predicted correlation )	Synthetic trajectories starting for real patients and entirely synthetic patients are generated. Missing data imputation is performed. CRBM does not model correlation between cognitive scores and other variables very well. Some crucial parameters, such as levels of amyloid, are omitted from the dataset. Overall performances of the model are significant
^55^	36 subjects (HC: 13, AD: 23) / 32 (HC: 8, AD: 24)	504 / 488	VBSD / Dem@Care	Extracted spectrogram features from subjects’voices. Each recording is previously segmented.	Train: 35/31Test: 1(subjects)	Logistic Regression CV (best among others)	Accuracy: 0.833 / 0.844 Precision: 0.869 / 0.913 Recall: 0.869 / 0.875 F1-Score: 0.869 / 0.894	It provides new and inventive approach for analyzing and predicting the disease. No data preprocessing is performed. Even after the segmentation, datasets are still small-sized. Hyperparameter tuning is not applied
^57^	343 sessions -150 subjects (ND: 72, D:78)	15	Open Access Series of Imaging Studies (OASIS)	MRI scans and other brain measurements, MMSE and CDR scores, demographic data	Random selection allocation for train, validate and test	Random Forest (best among others)	Accuracy: 0.868 Precision: 0.941 Recall: 0.8 AUC: 0.872	Detailed data processing and examination. Complete workflow following consecutive stages from data preprocessing to model evaluation. Only first visit for each patient is taken into account (e.g. cases when a patient convert from non-demented to demented are omitted). Only simple imputing techniques are considered
^59^	373 sessions–150 subjects (ND:72, D:64, C:14)	15	Open Access Series of Imaging Studies (OASIS)	MRI scans and other brain measurements, MMSE and CDR scores, demographic data	10-fold cross-validation	Hybrid modeling (combination of four models)	Accuracy: 0.980 Precision: 0.981 Recall: 0.980 ROC: 0.991	Three different approaches are being analyzed: manual feature selection, automatic feature selection and hybrid modeling. Results obtained by hybrid modeling are fascinating, containing high and stabile values. Not a single stage of data preprocessing and engineering is performed
^54^	5000 images (Mild, Very Mild, Non, Moderate Demented)	1700 region proposals per image	Alzheimer’s Disease Neuroimaging Initiative (ADNI)	MRI scan images	Separate datasets for train and test	SVM, R-CNN and Fast R-CNN	Training time (h): R-CNN: 84 Fast R-CNN: 8.75	The main goal is to provide comparison between different object detection algorithms in terms of their training and predicting times. No prediction results and accuracy metrics are shown. No data preprocessing is shown
^60^	1721 subjects (521 NC, 864 MCI, 336 AD)	47	AD Neuroimaging Initiative (ADNI)	MRI and PET scans, CSF, gene expression and cognitive scores	Train: 70 %Validate: 15% Test: 15%	Recurrent Neural Network	Accuracy:AD - NC: 0.959 AD - MCI: 0.859 NC - MCI: 0.773	Whole focus is put on the RNN algorithm its possibilities and its evaluation. Filling data between different timestamps is performed on three various approaches. No information about data preprocessing is given. No missing data imputation is performed (missing values are replaced with 0)
^11^	202 subjects (52 HC, 99 MCI, 51 AD)	189 (MRI ROI: 93, PET ROI: 93, CSF: 3)	Alzheimer’s Disease Neuroimaging Initiative (ADNI)	MRI, FDG-PET and CSF biomarkers	10-fold cross-validation	SVM (multiple kernel combination)	Accuracy: 0.932 Specificity: 0.933 Recall: 0.930	This study represents unified way of combining data from different sources into one kernel. Only three types of data are being used. An improvement of one model’s effectiveness using precise feature selection is shown. Before usage, images are preprocessed
^10^	Group I: CN:20, AD:20;Group II: CN:14,AD:14;Group III: CN: 57, AD: 33; Group IV: FTLD: 19	–	Each group of subjects comes from different community or research center	MR scans	Leave-one-out technique	SVM	Group I / Group II / Group III / Group IV: Accuracy:0.950 / 0.929 / 0.811 / 0.892 Specificity: 0.950 / 0.857 / 0.930 / 0.947 Recall: 0.950 / 1.00 / 0.606 / 0.833	Differentiation between AD and FTLD subjects is represented as they are often misidentified. Detailed image preprocessing is performed. Results are better than most of the scientific works that used MRI before. Only two diagnoses at a time are taken into classification
^53^	785 subjects ( 184 HC, 228 sMCI, 181 pMCI, 192 AD)	–	AD Neuroimaging Initiative (ADNI)	ROI, APOe4, cognitive scores and demographic data	10-fold cross-validation	CNN	Accuracy: 0.925 Specificity: 0.850 Recall: 0.875	Very detailed and mathematically supported approach of using neural networks for classification is presented. Data preprocessing and feature selection is performed. Special attention is put on avoiding over/underfitting problems. All data is baseline

Although findings and results obtained in scientific papers mentioned above cannot be directly compared with this research because of differences in approaches and data being used, still a general comparison can be provided. It can be noticed that instead of using data sets containing quantified values of different measurements (that is an example of our data set), some researchers use image processing of MRI scans^10,54^ and spectrograms of patients’ voices^55^. In each research paper patients are labeled with a particular diagnosis. Although in some, patients are only distinguished between two different clinical phases^10,55–57^. Comparing the obtained metrics from the prediction, it can be seen that most of the researches that use ADNI datasets managed to achieve impressive accuracy of their models, even greater than 0.9. The main goal in all these researches is to accomplishing the highest possible metrics in order to create a model that will predict a particular diagnosis most accurately and with greatest precision. Yet, it still remains unclear in which manner the model managed to achieve such results (i.e., the model is treated as a black-box without providing explanations about the contribution and influence of each feature to the end prediction). In this research, model’s metrics serve as a validation for the provided in-depth analysis and interpretability. Using explainable machine learning methods, a bigger picture about features influence and their correlation is being presented. The main goal in this research is not to create the best model (i.e., we are focusing only on a model with a good accuracy that can be used for providing interpretations), but rather to dive deeper into the importance and influence of each clinical measurement on a particular diagnosis.

Besides the satisfying metrics obtained from the model’s evaluation, clinical insights are also of a significant importance to validate the attained ranking of the features. The ranking follows the same manner as the disease symptoms progress from micro (based on cell metabolism and gene expression) to macro level (based on losing brain mass and cognitive decline)^58^. Gender and gene expressions are considered as predispositions, thus they do not represent strong and direct indications for the disease. On the other hand, results from cognitive tests provide clear and efficient understanding of the patient’s mental condition and clarify the exactness of it. The difference in importance between different types of scanning yet needs to be considered and validated from clinical experts.

## Conclusion

The main objective of the study presented is to put at test the existing hypothesis regarding the causes and indicators of Alzheimer’s disease. At the beginning of the research, four hypothesis were established considering the existing published literature. Hereafter, a large data set was obtained containing various types of features considering the lifestyle, personal information, medical analysis and cognitive tests of 12741 individuals (subjects). The data set was used in accordance with ethics and after obtaining a special permission for research goals.

To test the established hypothesis, intelligent models were built by following a ML approach. The high performance of the model (XGBoost) was used in advantage of explainable ML methods able to interpret the relations among the various features and therefore, to derive conclusion over the causes and indicators of the Alzheimer’s disease.

The most important contribution from this research is the established scheme presented in Table 8. This table provides a summarized features positive or negative influence on diagnosing each class, according to the global interpretability of both of the intelligent models built.

Thus, the comprehensive analysis of the features importance considering both the global and local interpretability, led to the following important conclusions regarding the previously established hypothesis:

- Cognitive tests, especially CDRSB, have greatest influence on one model’s outcome regarding all of the classes.
- Higher CDRSB values tend to correlate with more devastating diagnoses.
- Subject’s gender impact slightly on the model’s outcome.
- There is a unique combination of MRI indicators influence for each of the diagnosis, and this might be really interesting for the physicians.
- APOE gene is not always a decisive factor in determining a diagnosis.
- Important thing noticed is high and low education influence positively on determining the extremely distance diagnoses, CN and AD, correspondingly.
- It also can be noticed that results from cognitive tests greatly contribute into the false prediction.
- It can also be confirmed that subjects with lower values of FDG tend to be diagnosed with AD, but also with LMCI.
- Subjects with lower FDG tend to have higher CDRSB results.
- Fascinating is how the positive correlation that FDG and CDRSB have with the outcome gradually evolves into negative one, as the disease stages move from CN to AD.
- Younger subjects with two alleles e4 of APOE gene also have bigger chances of developing AD.
- High values for AGE and zero e4 alleles have negative impact on developing AD.
- One e4 allele and higher AGE values have a positive impact on developing AD.

Considering the conclusions, it can be stated that using a data-driven approach, all the hypotheses are being rejected, showing that AD is a complex disease that cannot be initiated by genetics alone, nor the gender, nor the age, nor the lack of education. It is important to note that conclusions obtained from data-driven interpretability, as in this case, cannot be taken for granted without consideration of medical experts, but they can provide significant hints and possible indications for further medical examinations and research.

This research is believed to have big influence on the future directions for understanding AD as well as large influence on the future researchers regarding the usage of explainable ML methods to unveil new knowledge also in other diseases data.

Such analysis are expected to affect also the medical approaches for on-time diagnosing and therefore, proper treating with the aim to slow down the progression of the disease, and thus reduce the damage that this disease causes to a person, as well as to his surrounding.

## Data Availability

Data used in the preparation of this article were obtained from the Alzheimer’s Disease Neuroimaging Initiative (ADNI) database (adni.loni.usc.edu). The ADNI was launched in 2003 as a public-private partnership, led by Principal Investigator Michael W. Weiner, MD. The primary goal of ADNI has been to test whether serial magnetic resonance imaging (MRI), positron emission tomography (PET), other biological markers, and clinical and neuropsychological assessment can be combined to measure the progression of mild cognitive impairment (MCI) and early Alzheimer’s disease (AD). For up-to-date information, see www.adni-info.org.
